# Combination of *In Silico* and *In Vitro* Screening to Identify Novel Glutamate Carboxypeptidase II Inhibitors

**DOI:** 10.1021/acs.jcim.2c01269

**Published:** 2023-02-17

**Authors:** Veronika Temml, Jakub Kollár, Theresa Schönleitner, Anna Höll, Daniela Schuster, Zsófia Kutil

**Affiliations:** † Department of Pharmaceutical and Medicinal Chemistry, Institute of Pharmacy, Paracelsus Medical University, Strubergasse 21, 5020 Salzburg, Austria; ‡ Laboratory of Structural Biology, Institute of Biotechnology of the Czech Academy of Sciences, BIOCEV, Prumyslova 595, 252 50 Vestec, Czech Republic

## Abstract

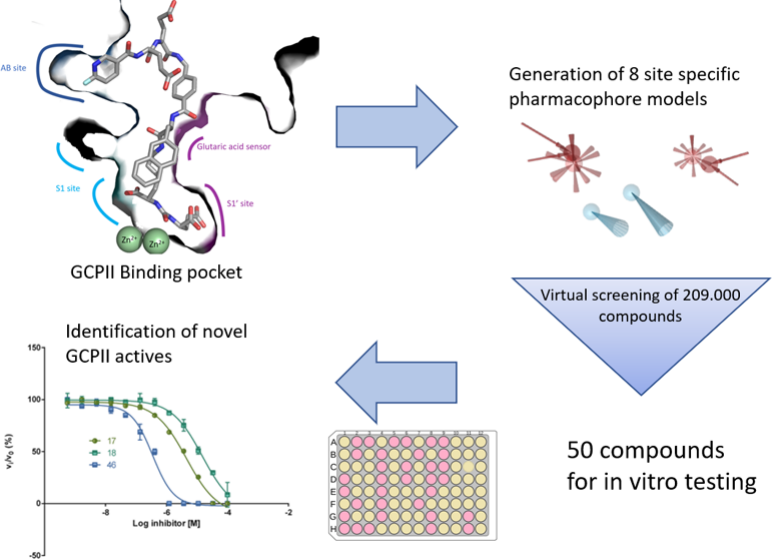

Glutamate carboxypeptidase II (GCPII) is a metalloprotease implicated in neurological diseases and prostate oncology. While several classes of potent GCPII-specific inhibitors exist, the development of novel active scaffolds with different pharmacological profiles remains a challenge. Virtual screening followed by *in vitro* testing is an effective means for the discovery of novel active compounds. Structure- and ligand-based pharmacophore models were created based on a dataset of known GCPII-selective ligands. These models were used in a virtual screening of the SPECS compound library (∼209.000 compounds). Fifty top-scoring virtual hits were further experimentally tested for their ability to inhibit GCPII enzymatic activity *in vitro*. Six hits were found to have moderate to high inhibitory potency with the best virtual hit, a modified xanthene, inhibiting GCPII with an IC_50_ value of 353 ± 24 nM. The identification of this novel inhibitory scaffold illustrates the applicability of pharmacophore-based modeling for the discovery of GCPII-specific inhibitors.

## 1 Introduction

Glutamate carboxypeptidase II (GCPII; a.k.a. *N*-acetyl-l-aspartyl-l-glutamate peptidase I, folate hydrolase, prostate-specific membrane antigen) is a membrane-tethered zinc-dependent metallopeptidase. GCPII is highly expressed in most prostate cancers, and even though its function in the pathology of prostate carcinoma is currently unknown, high expression levels of GCPII in this tissue are exploited for prostate cancer imaging and therapy. In the brain, the enzyme is primarily responsible for the hydrolysis of *N*-acetylaspartylglutamate (NAAG), a highly abundant peptide neurotransmitter, yielding *N*-acetylaspartate and glutamate. Under physiological conditions, the coordinated action of these molecules modulates neuron–neuron and neuron–glia communication. Under pathologic conditions, however, excessive glutamate levels can lead to neuronal dysfunction and degeneration, and glutamate excitotoxicity has been linked to various neurological disorders.^1^ GCPII is thus considered a promising target in this area. However, practical applications of inhibitors targeting GCPII within the neuronal compartment are limited by their unfavorable physicochemical characteristics and ADME profile. Further development of GCPII inhibitors capable of blood–brain barrier penetration is thus highly desired.

**Figure 1:**
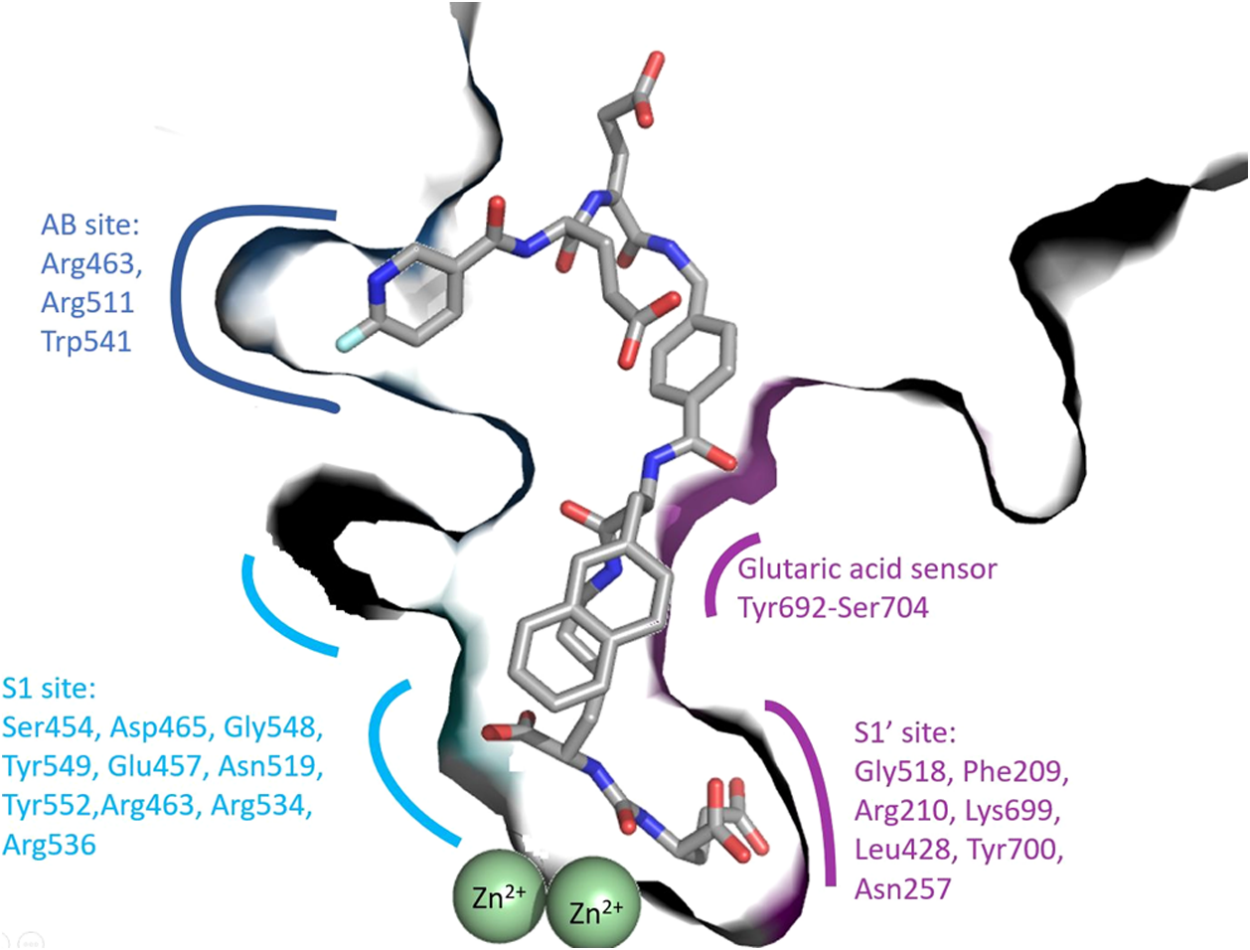
Structure of the GCPII/PSMA-1007 complex (PDB entry 5o5t ^21^) illustrates the anatomy of the GCPII internal cavity and the S1, S1′, and AB site (ABS) pockets. Zinc atoms are shown as green spheres. The S1 binding site consists of residues Ser454, Asp465, Gly548, Tyr549, Glu457, Asn519, Tyr552, and a positively charged arginine patch (Arg463, Arg534, and Arg536, pale cyan). The S1′-binding site (purple) is formed by residues Gly518, Phe209, Arg210, Lys699, Leu428, Tyr700, and Asn257. Residues 692–704 constitute a glutaric acid sensor. The arene binding site (ABS blue) is formed by Arg463, Arg511, and Trp541.

Detailed insights into structural features governing ligand recognition by GCPII can be used for the structure-assisted design of GCPII-specific compounds (reviewed in ref (2)). The primary site of substrate/inhibitor interactions with the enzyme is its internal cavity, which can be divided into the prime (S1′ pharmacophore pocket) and nonprime (S1 pocket and entrance funnel) regions. These two prominent segments of the internal cavity are separated by the active site harboring two zinc ions. The S1 pocket is a loosely defined spacious region that can accommodate a variety of moieties of diverse size, stereochemistry, and physicochemical characteristics.^3^ The only and most prominent structural motif that can be selectively targeted in this area is the positively charged arginine patch comprising Arg463, Arg534, and Arg536 side chains.^4^ Contrary to the ill-defined S1 pocket, the S1′ pocket binds glutamate and glutamate-like moieties of substrates/inhibitors with high selectivity and affinity. In reality, glutamate and glutamate-like functionalities are key motifs in inhibitors selectively targeting the S1′ pocket. Nevertheless, the majority of “canonical”, i.e., glutamate-containing inhibitors are highly charged, suffer from poor oral bioavailability, and their practical use for targeting GCPII residing in the neuronal compartment is strongly limited. An additional structural motif that can be exploited for the development of GCPII-specific inhibitors is the exosite at the entrance lid termed the arene binding site^5^ (Figure 1). The strategies of GCPII targeting can be roughly divided into the development of canonical and noncanonical inhibitors. The canonical inhibitors comprise a moiety derived from or mimicking the substrate linked to a zinc-binding group (ZBG). ZBGs, such as ureas, phosphorus-based functions, and hydroxamates, are critical for high affinity, while the glutarate part of the inhibitor is required for pronounced specificity against GCPII. The most developed canonical inhibitors are highly stable and soluble with a high affinity to GCPII. Potent examples of this inhibitor class are the urea-containing glutarates DCIBzL, with an IC_50_ of 0.06 nM^6^ and RNA 2-49-1 with an IC_50_ of 0.08 nM.^7^ Several other GCPII inhibitors with subnanomolar activities are described in the SI, Chapter 7 (Figures S20 and S21) and in the literature.^6−15^ Notably, they all share a common glutamate structure that is accommodated in the S1′ binding pocket and conjugated with different types of zinc-binding groups, either urea, phosphonate, thiol, or hydroxamate. The most potent inhibitors contain a urea or phosphonate group (Figure S20).

There are three FDA-approved GCPII inhibitors currently in use as prostate treatment imaging agents (Figure S21): piflufolastat F18, glutamic acid, and pendetide. Many more GCPII inhibitory agents are currently being investigated in clinical trials in relation to cancer.

The neurotherapeutic potential of GCPII inhibitors was first indicated by a study that used 2-PMPA to exert a neuroprotective effect after acute ischemic brain injury.^16,17^ After this finding, GCPII inhibitors were tested in a wide variety of animal models for neurotherapeutic effects (see refs (5, 18)), even leading to early clinical trials for the thiol-based GCPII inhibitor GPI5693 (2-MPPA) for CNS disorders.^19^

However, poor oral availability and minimal penetration in the nervous system caused by so far discovered ZBGs hinder further development and their use in clinical practice. Attempts to create brain-penetrable prodrugs have been conducted, but they have not yet led to clinical trials.^20^ The development of noncanonical inhibitors is focused on the substitution of the glutamate-derived binding module by structurally unrelated functions and targeting the nonprime region without compromising the affinity. Substituting the glutamate moiety and decreasing the number of charged functional groups within the inhibitor would lead to more lipophilic compounds. Furthermore, targeting the spacious S1 pocket would enable the incorporation of bulkier substituents into inhibitory scaffolds allowing for a search within a wider chemical space. Overall, such approaches can be exploited for the discovery of novel compounds targeting GCPII residents in the neuronal compartment with favorable ADMET properties in the future.

Pharmacophore modeling is a long-standing computational strategy to find novel ligands for known protein targets. By designing a pharmacophore, an abstracted three-dimensional (3D) pattern of physicochemical features (such as hydrogen bonds, ionizable functionalities, hydrophobic regions, aromatic interactions, and metal-binding groups), the crucial interactions between a protein and a small ligand molecule can be used to search for other molecules with similar binding geometry.^22−25^ Pharmacophore models are built based on 3D structural data (experimentally determined by X-ray crystallography or cryo-electron microscopy) in the structure-based approach and can also be calculated by aligning multiple known active molecules and extracting common features (ligand-based approach).^22,24,26^

Pharmacophore modeling enables the screening of very large compound databases to find novel scaffolds that display the known interaction pattern for a target.^23^ In this study, we aimed to represent all of the different known GCPII binding modes as pharmacophores and to use these models to identify new noncanonical GCPII binding scaffolds.

## 2 Materials and Methods

### 2.1 Dataset Curation and Preparation

A set of 54 GCPII ligands with known binding modes were compiled from the Protein Data Bank (PDB) database.^27^ The dataset was divided into five subsets according to the binding modes of the co-crystallized inhibitors. To test the selectivity of the resulting models, a dataset of decoys was generated. A decoy dataset contains random compounds assumed to be inactive for modeling purposes.^26^ The complete ChEMBL database (version 30)^28^ was downloaded, and all known GCPII active compounds were removed. Out of the remaining structures, a structurally diverse subset of 2681 compounds with physicochemical properties similar to the active molecules dataset was selected. For clustering and physicochemical property filtering, Pipeline Pilot 2019 Client (BIOVIA, San Diego) was used.

All databases were converted into multiconformational screening databases with LigandScout^29,30^ 4.08’s implemented Omega conformer generator^31,32^ using default “best” settings (calculating a maximum of 500 conformers for each structure).

### 2.2 Pharmacophore Model Generation

Pharmacophore models consist of different feature types representing either specific interactions with the protein or steric requirements:^29,33^ positively (PI) and negatively ionizable (NI) features, hydrogen-bond donor/acceptor features (HBD/A), hydrophobic contacts (HC), aromatic interactions (AI), and metal-binding features. In addition, the models also contain exclusion volumes (Xvols) that prohibit steric clashes of the molecule with the binding pocket.

All pharmacophore models were generated using LigandScout 4.08 (www.inteligand.com). Models were generated either based on a specific protein–ligand complex (structure-based) or via a 3D alignment of a training set of active compounds (ligand-based). For creating ligand-based models, the shared feature mode was used, which results in a model containing features that are present in all training compounds.

Automatically generated pharmacophore models profit from manual refinement and optimization by altering feature sizes, removing selected features, and adapting or removing Xvols.^34^ Therefore, all pharmacophore models were further refined by repeatedly screening the set of active molecules and decoys and manually adapting the models to map the maximum number of active molecules in that specific binding mode and a minimal number of decoys.

To evaluate the discriminatory power of the models, several quality metrics were calculated for each model: sensitivity (eq 1),^35^ specificity (eq 2),^35^ yield of actives (YoA, eq 3),^36^ enrichment factor (EF, eq 4),^36^ accuracy (eq 5),^37^ and the receiver operating characteristic (ROC) curve,^35,38^ which can be summed up by the area under the curve (AUC).^26,35,39^
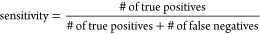
1
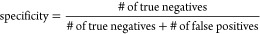
2
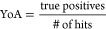
3
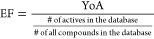
4
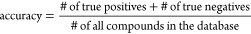
5

### 2.3 Prospective Screening

The generated pharmacophore models (models 1–8) were used to screen the SPECS database of commercially available synthetic compounds. All 208,968 compounds were downloaded from the website (www.specs.net, Specs_SC_10mg_April2021) and prepared for virtual screening. A 3D multiconformational database was created using the Omega conformer generator with default “fast” settings (calculating a maximum of 25 conformers).

### 2.4 Hit Selection

Virtual hits resembling the natural GCPII substrate (containing a glutamate moiety) were removed since they cannot be considered novel. The remaining hits were visually assessed for structural similarities considering that as many models as possible should be represented in the experimental validation. Therefore, hits from models that found only fewer than 10 hits were all selected for experimental testing. Finally, 50 compounds out of 82 virtual hits were experimentally evaluated.

### 2.5 *In Vitro* Inhibition Assay, IC_50_ Values, and Inhibition Mode

The inhibitory potency of the selected compounds against GCPII was determined using a fluorescence-based assay developed in-house. Recombinant human GCPII purified as described previously^40,41^ (final concentration 0.02 nM) was preincubated with 20 μM test compound for the screening in 50 mM Tris-HCl, 150 mM NaCl, 0.001% C_12_E_8_ at 37 °C for 10 min. The reaction was initiated by the addition of 100 nM Glu-Glu dipeptide labeled with fluorescein (substrate)^42^ in a total volume of 50 μL. Following 15 min incubation, the reaction was terminated by the addition of 5 μL of 0.1% TFA in 5% acetonitrile. Reaction mixtures were then analyzed by RP-HPLC with a Kinetex 2.6 μm XB-C18 100 Å column with a fluorescence detector set to λ_EX_/λ_EM_ = 492/516. The GCPII inhibition in the SPECS hits samples was calculated using the noninhibited reaction as a control. Inhibition constants of the compounds active in the *in vitro* screening were determined using increasing concentrations of inhibitors. The data were fitted using the GraphPad Prism software, and IC_50_ values were calculated from the inhibition curves of two independent experiments using a nonlinear analysis protocol.

To determine the mode of inhibition, the IC_50_ values of the compounds were measured in a separate experiment using the conditions described above but with the concentration of the substrate 10x above the *K*_M_ of the substrate (i.e., 1 μM Glu-Glu dipeptide labeled with fluorescein).

### 2.6 Inhibitor Specificity (HDAC10)

The specificity of the active compounds was evaluated by the determination of IC_50_ values against HDAC10, a zinc-dependent metallohydrolase unrelated to GCPII. The fluorescence-based activity assay used was developed in-house. Recombinant human HDAC10 purified as described previously^43^ (0.5 nM) was preincubated with dilution series of compounds in 50 mM 4-(2-hydroxyethyl)-1-piperazineethanesulfonic acid (HEPES), 140 mM NaCl, 10 mM KCl, 2 mg/mL bovine serum albumin (BSA), and 1 mM tris(2-carboxyethyl) phosphine, pH 7.4 at 37 °C for 10 min. The reaction was initiated by the addition of a 10 μM substrate (spermidine labeled with fluorescein, provided by the group of Prof. Mike Schutkowski)^43^ in a total volume of 50 μL. Following 30-min incubation, the reaction was terminated by the addition of 5 μL of 0.5% acetic acid and centrifuged at 2000*g* at room temperature for 15 min to remove precipitated BSA. Reaction mixtures were then analyzed by reversed-phase high-performance liquid chromatography (RP-HPLC) with a Kinetex 2.6 μm XB-C18 100 Å column with a fluorescence detector set to λ_EX_/λ_EM_ = 492/516. The HDAC10 inhibition was calculated using the noninhibited reaction as a control. Inhibition constants of the compounds were determined using increasing concentrations of inhibitors. The data were fitted using the GraphPad Prism software,^44^ and IC_50_ values were calculated from the inhibition curves of two independent experiments using a nonlinear analysis protocol.

## 3 Results and Discussion

### 3.1 Workflow

**Figure 2:**
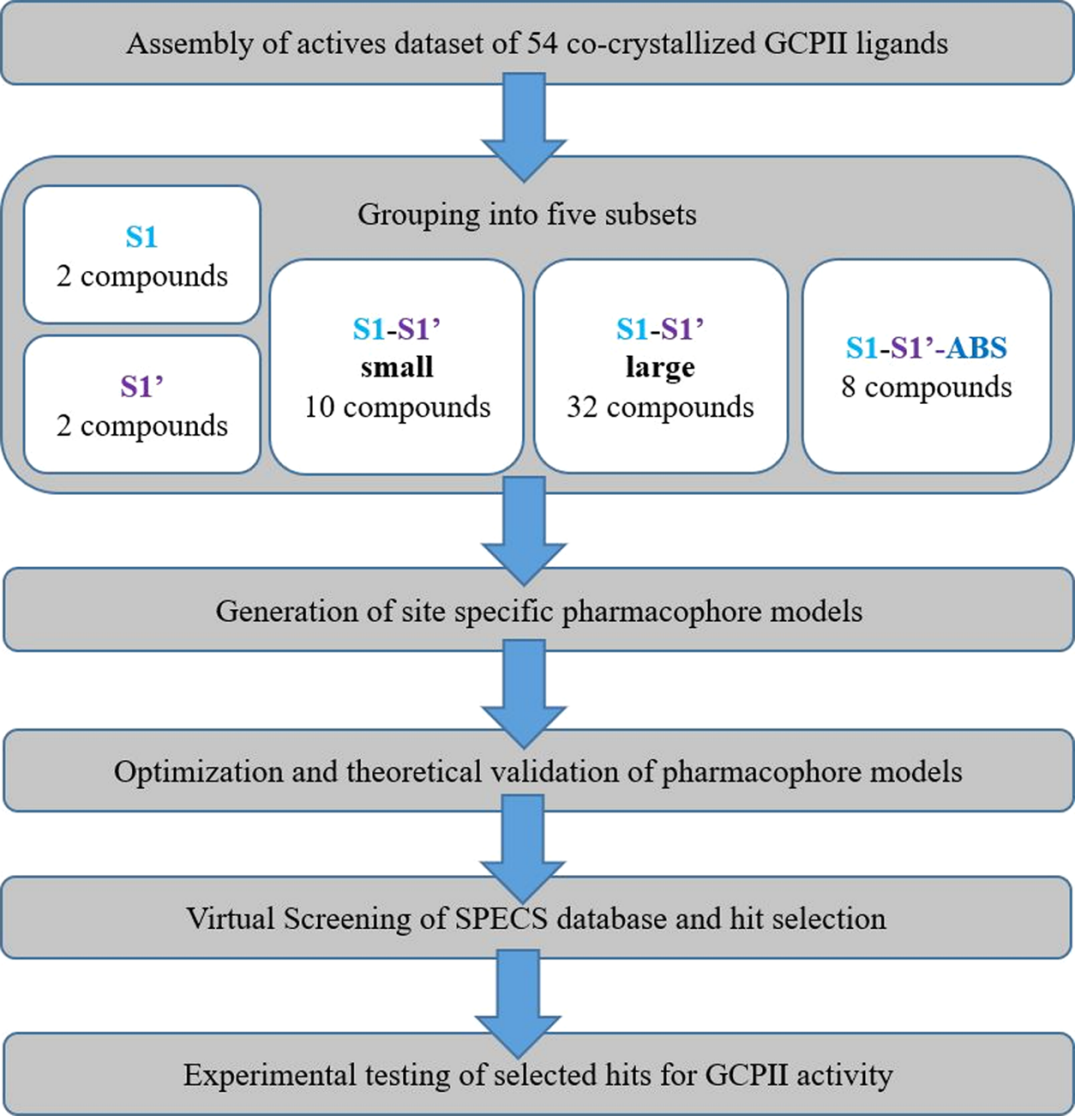
General workflow for the pharmacophore model-based search for novel GCPII inhibitors.

The general workflow applied in this study is summarized in Figure 2. The PDB database was searched for ligand-binding complexes of GCPII. A total of 54 compounds were integrated into a dataset of active ligands. The ligands were then categorized according to their binding modes and sizes into five subsets denoted (i) S1, (ii) S1′, (iii) S1&S1′ small, (iv) S1&S1′ large, and (v) S1, S1′,&ABS. Pharmacophore models were created for each binding mode and optimized to find as many actives and as few decoys as possible. During the optimization process, features were checked individually for their ability to enrich active compounds over decoy compounds. If a feature prohibited the model from finding actives but did not help to exclude decoys, it was removed. Distance and angle restrictions from the automatically generated models were also altered to include more active compounds. A detailed description of the optimization process for each model is given in the Supporting Information (SI).

### 3.2 Dataset Curation

The wealth of crystallographic data available for GCPII allowed us to assemble not only active compounds with known binding affinities but also active compounds with known binding modes. To capitalize on this rare knowledge, we aimed not only to generate models to generally predict GCPII activity but also to design site-specific pharmacophore models representing different prominent binding modes as well. The complete dataset of 54 active compounds is shown in Figures S1–S5. It was divided into five subsets based on the overall size of the compound and the GCPII pocket it occupies: (i) only two small compounds bound exclusively to the S1 site (subset S1, Figure S1) and (ii) two compounds were also found exclusively binding to the S1′ site (Figure S2); (iii) the majority of compounds bound to both the S1 and S1′ pockets, where 10 smaller ligands bound primarily to S1′ but also interacted with parts of S1. These were grouped into the S1 and S1′ small datasets (Figure S3); (iv) the largest dataset (32 compounds) comprised larger molecules that bound to both the S1 and S1′ binding sites (Figure S4); and finally, while there were no compounds that bound solely to the ABS, eight large compounds spanned all three bonding pockets (S1, S1′, and ABS, Figure S5).

To evaluate the selectivity of the pharmacophore models, a dataset of 2681 structurally diverse compounds with physicochemical properties similar to the active molecules was created from the ChEMBL database^28^ as a decoy set.

### 3.3 Pharmacophore Model Generation and Optimization

For each binding mode, at least one structure-based pharmacophore model was generated. A total of eight pharmacophore models were created. The automatically generated models represent the interactions between the co-crystallized ligand and the protein. To create a model with the ability to find multiple similarly bound compounds, it has to be manually optimized. The models were improved by removing features, adding or removing Xvols, and altering feature tolerances to find a maximum of active molecules and a minimum of decoys. In case not all actives were found after the optimization process, an additional ligand-based model was created by aligning the compounds that were missed by the structure-based model. Detailed descriptions and depictions of the models can be found in SI Section 2.

**Figure 3:**
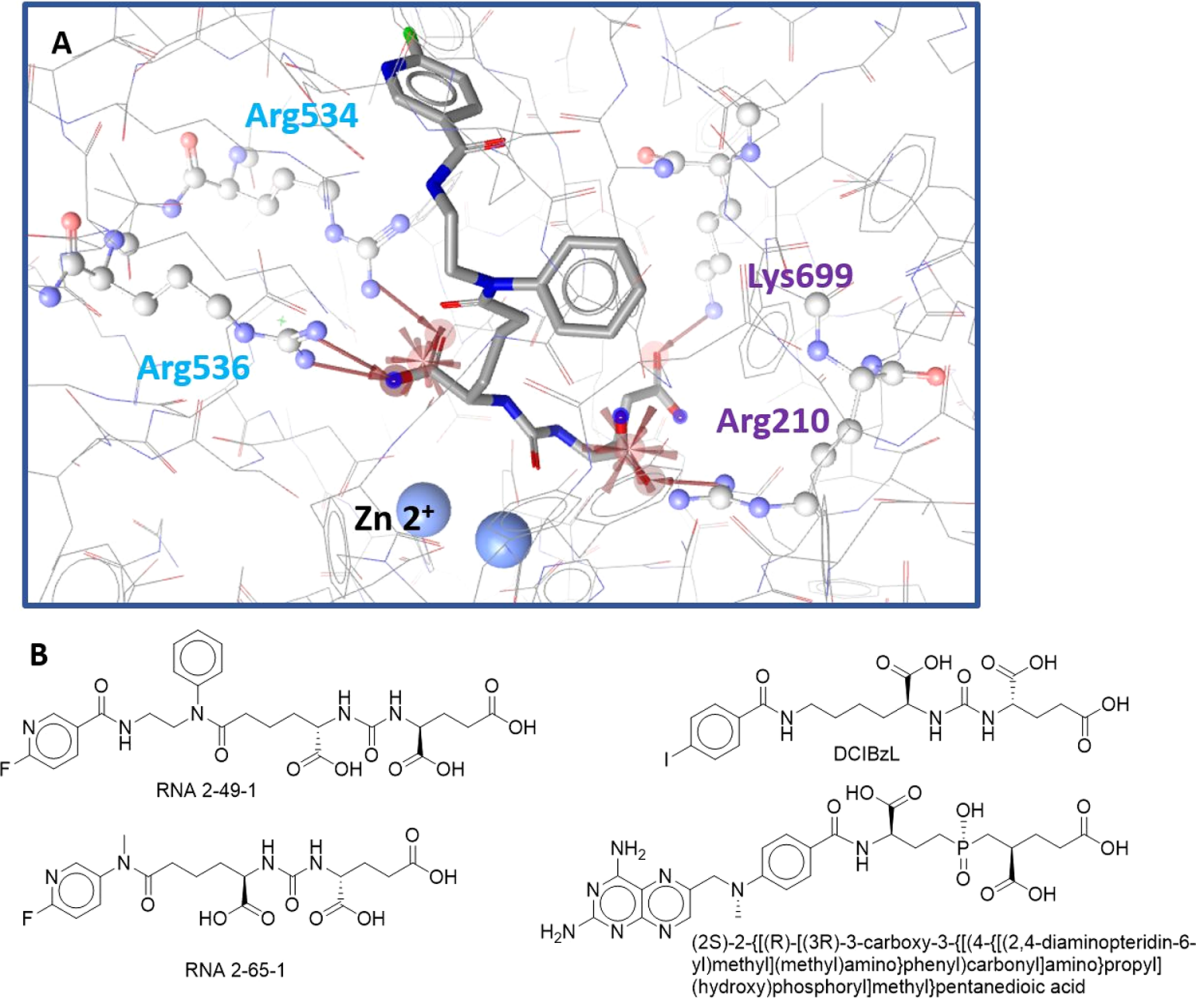
(A) Model 4: Optimized, shared-feature structure-based pharmacophore model for the S1–S1′ large subset with ligand RNA 2-49-1 (PDB entry 6HKZ ^7^). (B) Structures of ligands used to generate model 4.

Model 1 was generated based on two enantiomeric inhibitors (JHU241 and JHU242) binding to the S1 site (PDB 5D29 and 5ELY)^45^ (Figure 3B). Models for both structures were generated and combined into a shared feature pharmacophore model using the coordinates of 5ELY.^45^ This model was highly restrictive (EF = 1341.5, maximum value) and only found the two compounds in the dataset.

Two models were generated for the S1 and S1′ small subsets comprising 10 different ligands (Figure S3):

Model 2 (Figure S8) was generated based on three inhibitors, namely (2S)-2-[(*N*-acetyl-l-α-aspartyl)amino]nonanoic acid (PDB entry 3SJE(46)), (2*S*)-2-[(*N*-acetyl-l-α-aspartyl)amino]octanoic acid (3SJG ^46^), and *N*-acetyl-aspartyl-methionine (3SJX ^46^). The model correctly identified 10 out of 12 actives for this binding site and 40 decoys. The enrichment metrics are shown in Table 1. This model was less restrictive than model 1 and covered mainly smaller molecular entities.

**Table 1:** Quality Metrics of Pharmacophore Models^a^

model	1	2	4	5	6	7	8	all
TP	2	10	29	20	31	8	8	52
FP	0	40	21	10	36	14	4	78
TN	2681	2641	2660	2671	2645	2667	2677	2603
FN	0	2	3	12	1	0	0	2
# of actives in the database	2	12	32	32	32	8	8	54
decoy hitrate (%)	0.00	1.49	0.78	0.37	1.34	0.52	0.15	2.91
accuracy	1.00	0.98	0.99	0.99	0.99	0.99	1.00	0.97
YoA	1.00	0.20	0.58	0.67	0.46	0.36	0.67	0.40
EF	1341.50	44.88	49.17	56.52	39.23	122.23	224.08	20.26
EF_max_	1341.50	224.42	84.78	84.78	84.78	336.13	336.13	50.65
EF/EF_max_	1.00	0.20	0.58	0.67	0.46	0.36	0.67	0.40
sensitivity	1.00	0.83	0.91	0.63	0.97	1.00	1.00	0.96
specificity	1.00	0.99	0.99	1.00	0.99	0.99	1.00	0.97
AUC	1.00	0.91	0.95	0.81	0.98	1.00	1.00	0.97

a Only actives within the specific binding mode were considered when calculating the parameters. The database of decoys was composed of 2681 compounds (TP, true positive; FP, false positive; TN, true negative; FN, false negative; YoA, the yield of actives; EF, enrichment factor; AUC, the area under the curve).

An additional pharmacophore model (model 3) was created to cover the two remaining inhibitors of the S1′ site, but this model did not add any extra value to the model collection during the validation process. Consequently, it is only described in the SI and was not part of the experimental validation.

The largest part of the dataset (32 compounds) was assigned to the S1 and S1′ large subsets. A structure-based model (model 4) and two ligand-based models (models 6 and 7) were generated to recover all actives in this dataset.

Model 4: This structure-based model was created for the S1–S1′ large subset, containing 32 molecules (Figures S4 and S11), and covered interactions with both pockets of the internal cavity. It was based on the four most active inhibitors from the dataset: RNA 2-49-1, RNA 2-65-1, DCIBzL, and (2*S*)-2-{[(*R*)-[(3*R*)-3-carboxy-3-{[(4-{[(2,4-diaminopteridin-6-yl)methyl](methyl)amino}phenyl)carbonyl]amino}propyl](hydroxy)phosphoryl]methyl}pentanedioic acid, from PDB entries 6HKZ,^7^6H7Z,^7^3D7H,^6^ and 3BI1, ^8^ respectively. The combined, optimized model consisted of seven features and 19 Xvols. Two NI features anchor ligands on both the S1 and S1’ sides of the internal cavity. On the S1 side, the NI bonded to the catalytic Zn^2+^ ion and was supported by HBA interactions with the arginine patch (Arg534 and 536). The second NI bonded to Arg210 and was also supported by HBAs interacting with Arg210 and Lys699 (Figure 3). The model found 29 out of the 32 active molecules and mapped 21 decoys (Table 1). This model covered the largest part of the GCPII inhibitor’s active space since it found compounds that bind to the region close to the Zn^2+^ ions.

In addition to the structure-based model, two ligand-based models (models 5 and 6) were created for this subset. The enrichment metrics of both models are shown in Table 1, and the optimization process is detailed in the SI.

Finally, the subset spanning S1, S1′, and ABS encompassed eight very large inhibitors (Figure S5). Structure-based model 7 was based on the co-crystallized inhibitor ARM-P4 and consisted of a total of 9 features and 29 Xvols (Figure S15A). The model found all eight inhibitors in the training set and 14 decoys, leading to an EF of 122.2 (Table 1).

Since this structure-based model did not have any interaction features in the ABS part of the pocket, an additional ligand-based model was created based on all eight structures in the subset. Model 8 consisted of four HBA features and an NI feature that are spread over a distance of more than 15 Å (Figure S17). Due to its size, model 8 is highly selective for the S1, S1′, and ABS subsets compared to the structure-based model and found only four decoys while retrieving all actives from this subset (Table 1).

### 3.4 Experimental Validation through Prospective Virtual Screening

Models 1, 2, 4, 5, 6, 7, and 8 were used to screen the SPECS database (Specs_SC_10mg_April2021). Model 1 found no virtual hits in the SPECS database due to its very high restrictivity (EF = 1341.5, Table 1) and therefore could not be experimentally validated. Cumulatively, the remaining models identified a total of 82 virtual hits. Out of these, 23 hits were found by more than one model (an overview of the consensus hits is given in Table 2). The number of hits found by an individual model in the SPECS database correlated with their calculated restrictivity, with the highly restrictive models (models 8 and 9) only finding two compounds each and the fairly general model 6 mapping 51 hits (Table 3).

**Table 2:**
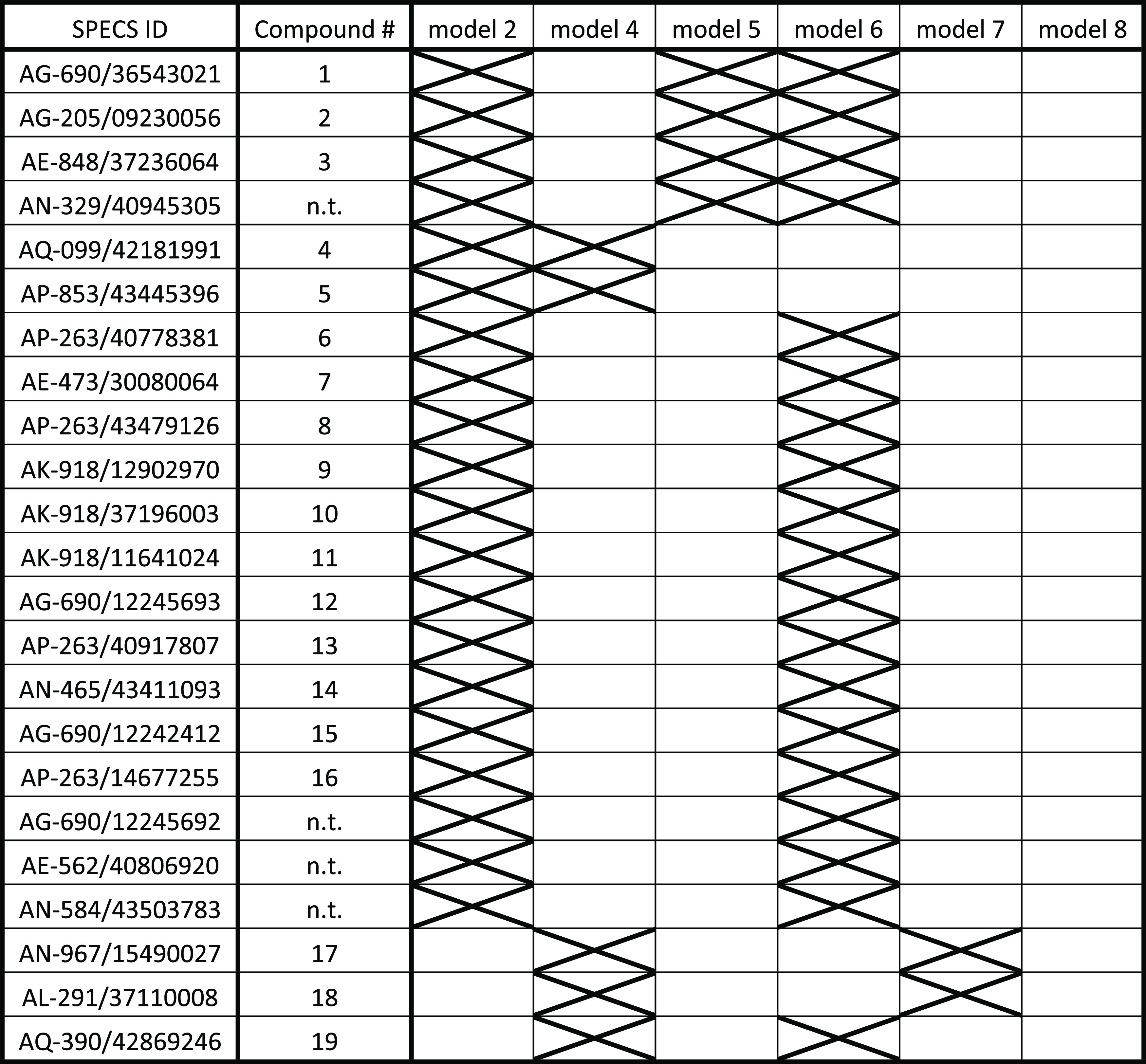
Overview of the 23 Virtual Hits from the SPECS Database That Mapped More Than One Pharmacophore Model^a^

a nt, not tested. 19 hits were experimentally tested (see compound #) *in vitro* on their GCPII inhibitory potency. The X marks models that mapped the compound.

**Table 3:** Number of Hits Per Model in SPECS Database^a^

model	# of SPECS hits	Consensus hits	unique hits	EF	accuracy
1	0	0	0	1341.50	1.00
2	34	20	14	44.90	0.98
4	15	5	10	49.2	0.99
5	5	4	1	56.5	0.99
6	51	19	32	39.2	0.99
7	2	2	0	122.2	0.99
8	2	0	2	224.1	1
all models	82	23	59	20.26	0.97

a Specs_SC_10mg_April2021.

Out of the 82 hits, 50 were selected for experimental testing. Compounds containing a glutamate-like moiety were removed due to structural similarity to known GCPII-inhibiting compounds. Among highly similar compounds, only one was selected for experimental testing. A detailed description of the filtering process and the structures of all tested compounds are shown in SI Section S3.

### 3.5 Experimental Validation of Inhibitory Potency of Selected SPECS Hits

**Figure 4:**
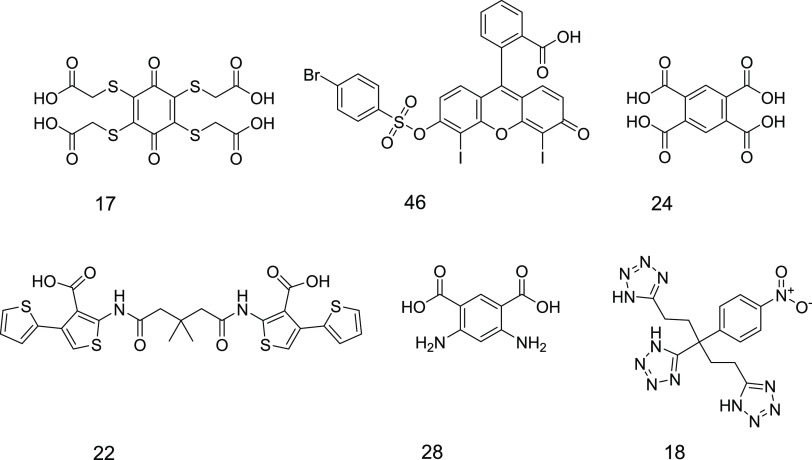
Structures of the active virtual hits found in the screening.

To experimentally validate the *in silico* data, we evaluated 50 selected virtual hits (Figure S13) *in vitro* in an enzymatic activity assay using highly purified human recombinant GCPII and the fluorescently labeled Glu–Glu dipeptide as a substrate. The results of the preliminary inhibitor screening at a concentration of 20 μM are detailed in Table S2. Three compounds showed more than 50% inhibition, namely, compounds **17**, **18**, and **46** (Table S2). Additional three compounds showed modest inhibition (>25%), namely, compounds **22**, **24**, and **28** (Figure 4).

**Figure 5:**
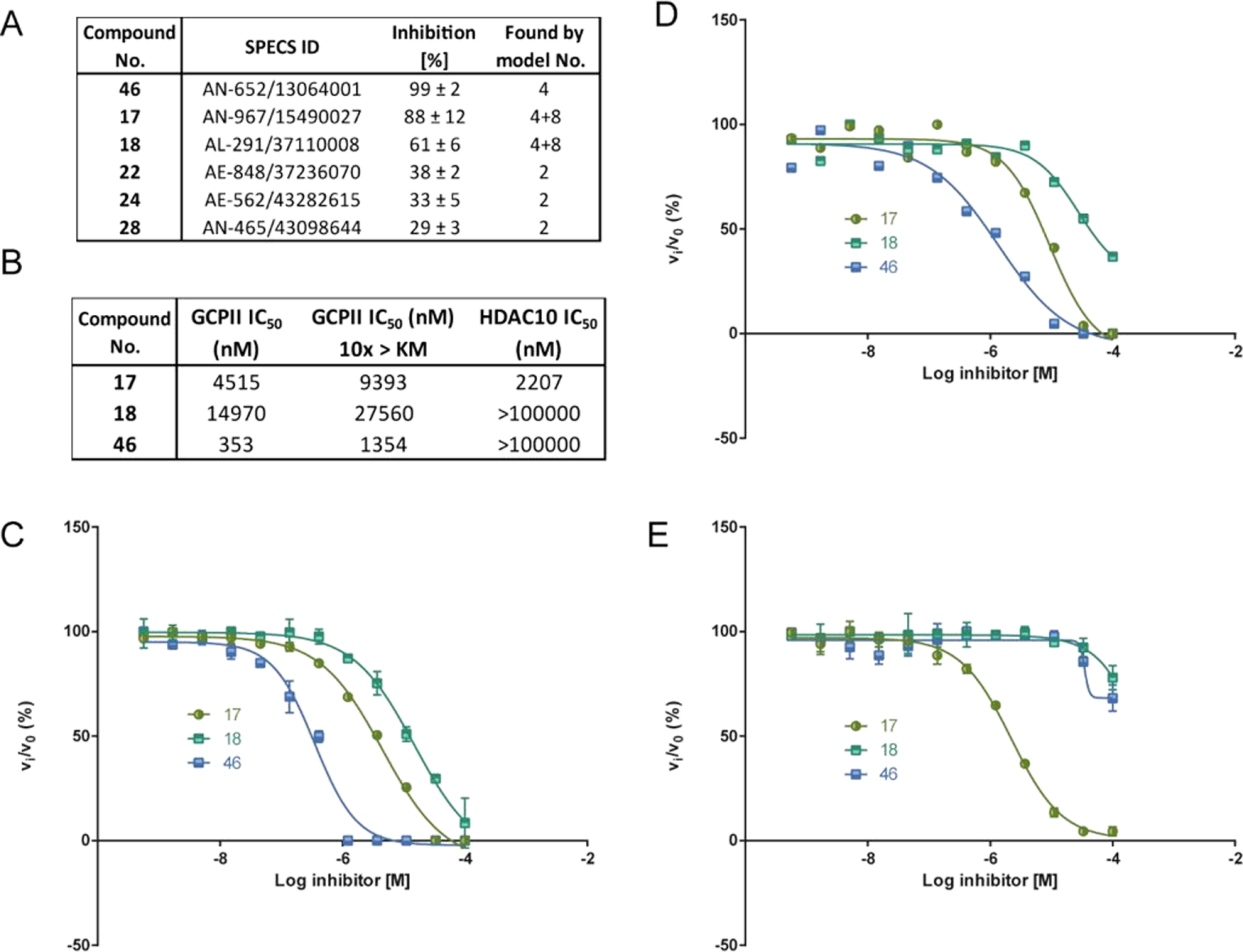
Enzymatic profiling of selected hits. (A) Inhibitory potency of the active compounds against GCPII. (B) IC_50_ values of the three most potent compounds for GCPII, and substrate concentration at *K*_M_, GCPII and substrate concentration 10 times above the *K*_M_, and against HDAC10. The respective full-dose–response inhibition curves are presented in panels (C), (D), and (E).

For the three virtual hits showing the highest GCPII inhibitory potency (compounds **17**, **18**, and **46**), IC_50_ values were determined. Compound **46** was found to be the most potent GCPII inhibitor tested with an IC_50_ of 350 ± 24 nM. Compounds **17** and **18** inhibited GCPII activity in the micromolar range, with IC_50_ values of 4.5 ± 0.1 and 15± 8.6 μM, respectively (Figure 5). All three most active compounds were identified by Model 4. Compounds **17** and **18** were identified as double consensus hits of models 4 and 7 and were the only hits of Model 7. The weakly active compounds **22**, **24**, and **28** were all identified as unique hits of Model 2. Models 5, 6, and 8 could not identify any active compounds.

To assess the type of GCPII inhibition, the IC_50_ values of the three most potent GCPII inhibitors were remeasured using a substrate concentration 10 times above the *K*_M_ value. The IC_50_ values of compounds **17** and **18** increased approximately twice, to 9.4 ± 0.1 μM and to over 20 μM, respectively, and the IC_50_ for compound **46** increased 4-fold to 1.4 ± 0.1 μM. These results indicate a competitive mode of GCPII inhibition. As a positive control, we used DCIBzL and measured an IC_50_ of 0.05 ± 0.018 nM, which is in good agreement with previously published data.^6^

To determine the specificity of our hits for GCPII, we have determined their inhibitory potency against histone deacetylase 10 (HDAC10), a zinc-dependent hydrolase unrelated to GCPII that we selected as a potential off-target. Compound **17** inhibited HDAC10 activity with an IC_50_ of 2.2 ± 0.1 μM, comparable to GCPII inhibition, while compounds **18** and **46** did not exhibit any HDAC10 inhibition, confirming their GCPII specificity (Figure 5).

**Figure 6:**
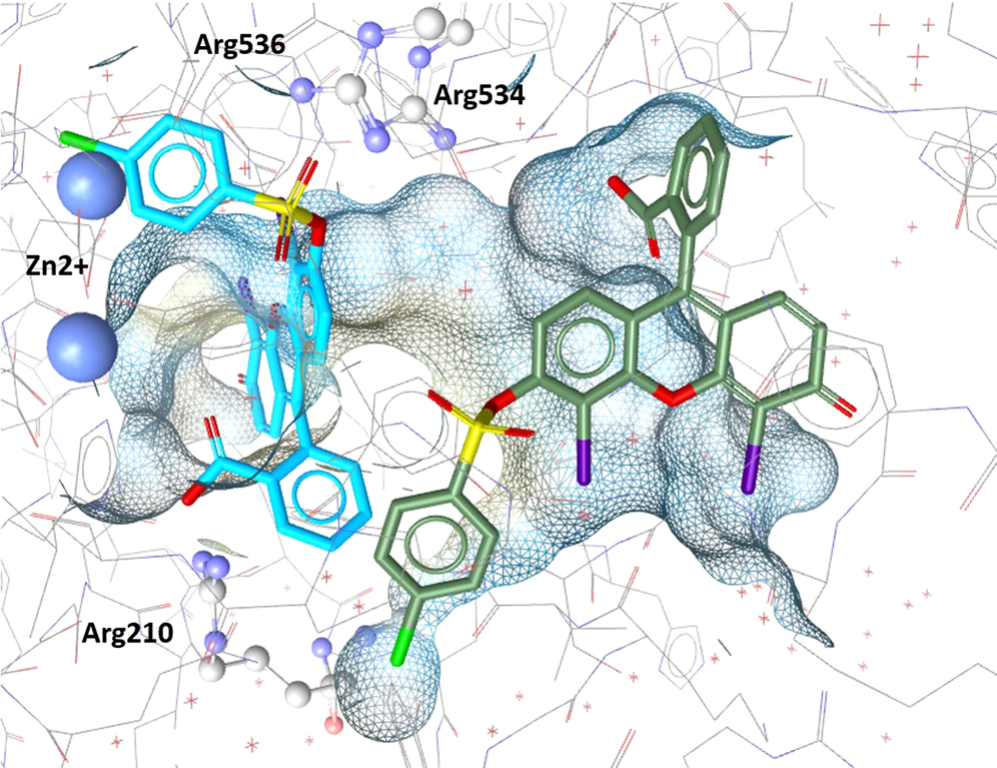
Suggested binding modes of compound **46** within the GCPII binding pocket. The binding mode based on the alignment of 46 with Model 4 is shown in blue; the binding mode proposed by the docking simulation is shown in green.

The superposition of compound **46** and model 4, which retrieved the hit, within the binding pocket revealed a steric clash with the active site Zn^2+^ ions (Figure 6, blue). It was therefore considered that the compound might bind in a similar binding mode farther away from the Zn^2+^ atoms. It has been shown in molecular dynamics simulations on other targets, e.g., COX-1,^47^ that compounds move through different energetic minima on their way into a binding pocket. Therefore, it is not uncommon to find similar binding motifs on the same protein. A docking simulation revealed a binding mode for compound **46** located closer to the opening of the binding cavity of GCPII (Figure 6, green, detailed description SI Section S6).

## 4 Discussion and Conclusions

Out of the eight pharmacophore models created in this study, six were used to retrieve virtual hits that were biologically tested for their inhibitory potency against GCPII. Model 1 and model 3 were created to cover the full range of known GCPII active chemical space but were only optimized to find two compounds. They were therefore too restrictive to retrieve hits for experimental validation. Among the other, six experimentally validated models, model 4, a structure-based model representing the S1 & S1′ binding modes, showed the best performance. It found three GCPII active compounds, including compound **46**, which was the most potent hit of the series with an IC_50_ of 353 ± 24 nM. None of the retrieved actives represent typically druglike molecules. However, this fact is not surprising, since the known GCPII actives are also large and charged at multiple positions. Compound **46** (Figure 4) is a xanthene scaffold modified with a sufonylbromophenyl, a benzoic acid, and two iodine moieties. This scaffold has no previous record of showing inhibitory potency against GCPII. Even though its potency is moderate compared to many known GCPII inhibitors in the low nanomolar or even picomolar range, the atypical structural features of the scaffold might be beneficial if it were used as a lead compound against this well-explored target.

Both actives found by structure-based model 7, the model representing the S1, S1′-ABS binding mode, were active in the micromolar range. However, the interpretation of their activity should be addressed with caution. Compound **17**, a cyclohexadione modified with four sulfanyl acetic acid moieties, also inhibited HDAC10 in an *in vitro* enzymatic assay and is a quinone-based compound. This scaffold was previously described to lead to false-positive readouts due to their interference with many activity assays (PAINS compound, see SI Section S5).^48^ Compound **18**, a nitrophenyl modified with three tetrazole rings, seems to be another selective GCPII inhibitor with a new active scaffold. The performance of the model is fairly impressive since it found 100% actives and can therefore be considered an excellent model to find GCPII binding ligands in future studies. Both hits were also partially mapped by Model 4. Model 2, a structure-based model for the S1′ binding mode, identified three weakly active compounds that, following further SAR optimization, could become more potent GCPII inhibitors. Compound **24** is a highly polar compound due to the presence of four carboxyl functions on a benzene ring. Compound **28** shares a similar structure with compound **24** having two carboxyl and two amino substitutions on its benzene ring. Compound **22**, though active only in the micromolar range, presents a new symmetric scaffold previously unreported for GCPII inhibitors and therefore bears a potential for future optimization. Nevertheless, any optimization efforts should be wary of the 2-amino-3-carbonylthiophene group that was shown to be protein reactive and cause protein thiol oxidation.^49^ This might, but need not necessarily, raise concern since this group rather rarely triggers nonspecific, false-positive readouts in activity assays.^48^ Also, GCPII does not contain any thiol group within its active site or in close vicinity that could act as a reaction partner. Compound **22** bears an additional aromatic substitution at position 4, and specific inhibitors of other proteins bearing this functional group exist, e.g., inhibitors of tubulin assembly,^50^ inhibitors of tyrosine kinase FLT3,^51^ or hepatitis C virus inhibitors.^52^

Out of the four binding modes, only the S1′ binding mode could not be experimentally validated because the model was too restrictive to find unique or new hits.

Ligand-based models 5, 6, and 8 did not identify any GCPII-inhibiting compounds. This illustrates that for this target, the structure-based approach vastly outperforms the ligand-based approach. This is likely due to the size of compounds and the binding pocket that represents very strict steric confinement. The binding pharmacophore can therefore not be found only by aligning active molecules without also providing the steric restrictions of the pocket.

Several GCPII inhibitors with activities in the subnanomolar range were reported in the literature.^6−15^ The compounds presented in this paper, albeit not reaching such impressive ranges of inhibitory activities, present new scaffolds previously unreported for GCPII inhibition. The highest Tanimoto similarity^53^ calculated using ECFP4 fingerprint^54,55^ between any of the reported compounds and known experimentally tested GCPII inhibitors is 0.256. For compound **46** specifically, it is only 0.156, indicating no similarity. Compounds **22** and **46** are also less polar than currently known GCPII inhibitors. The presented compounds can therefore serve as leads for optimization efforts in searching for derivatives with increased GCPII affinities and the ability to pass the BBB.

This study likewise illustrates how pharmacophore-based modeling can be successfully used to find novel GCPII active scaffolds that could serve as drug leads. The novel GCPII activities we report here are still very polar and not likely to pass the BBB. At the same time, however, such polar scaffolds could be effectively modified to create prodrugs for targeting GCPII residing in the neuronal compartment.^56^

## Data Availability

All datasets used within this work are available as sd-files as supporting files to this publication. For the generation and optimization of pharmacophore models as well as the prospective screening of the SPECS database, LigandScout 4.08 was used. A trial version of this commercial software valid for one month can be acquired from inte:ligand using the following link: http://www.inteligand.com/cgi-bin/ligandscout4/register.pl. Schrödinger commercial software package was used for the docking. The trial license can be acquired upon request by filling in the following form: https://www.schrodinger.com/request-sales-information. Canvas application from Schrödinger software package was used to screen for PAINS compounds and can be acquired using the above-mentioned link for Schrödinger. The database of readily commercially available compounds SPECS can be downloaded upon creating an account at www.specs.net website in the download databases section or under https://www.specs.net/index.php?view=databases&page=download. The database of compounds available at 10 mg was screened. Pipeline Pilot data analytics software (used for decoys generation) is commercial software that can be acquired online using the 3DS website https://www.3ds.com/products-services/biovia/products/data-science/pipeline-pilot/ and choosing the contact us option.

## Supporting Information Available

Detailed descriptions and structures of the datasets used in this study, a more in-depth description of the individual pharmacophore models and the optimization process, and additional experimental details (PDF)
Pharmacophore models and training datasets (ZIP)

## Abbreviations

ABS: arene binding site in the GCPII binding pocket
AUC: area under the curve
EF: enrichment factor
GCPII: glutamate carboxypeptidase II
HBA: hydrogen bond acceptor
HBD: hydrogen bond donor
HC: hydrophobic contact
NI: negatively ionizable
ROC: receiver operating characteristics curve
YoA: yield of actives
ZBF: zinc-binding feature
