# Supplementary material for: Combination of In Silico and In Vitro Screening to Identify Novel Glutamate Carboxypeptidase II Inhibitors
Source: J Chem Inf Model. 2023 Feb 17;63(4):1249–59. doi: 10.1021/acs.jcim.2c01269 (PMC9976286; doi:10.1021/acs.jcim.2c01269)
Supplement: Supplementary file 1 — ci2c01269_si_001.pdf [file ci2c01269_si_001.pdf]

Supporting Information

Combination of in silico and in vitro screening to identify novel glutamate carboxypeptidase II inhibitors

Veronika Temml[1], Jakub Kollár[1], Theresa Schönleitner[1], Anna Höll[1], Daniela Schuster[1], Zsófia Kutil[2]*

[1] Department of Pharmaceutical and Medicinal Chemistry, Institute of Pharmacy, Paracelsus Medical University, Strubergasse 21, 5020 Salzburg, Austria

[2] Laboratory of Structural Biology, Institute of Biotechnology of the Czech Academy of Sciences, BIOCEV, Prumyslova 595, 252 50 Vestec, Czech Republic

**Veronika.Temml@pmu.ac.at*

1. *Additional Information on Pharmacophore model generation*

All the pharmacophore models described in this paper were generated using LigandScout version 4.08 [1] as shared feature models. Shared feature structure-based models were created as follows. First, several structure-based models were generated based on corresponding GCPII-ligand crystal structures obtained from PDB database [2]. The individual structure-based models were overlaid on each other and a shared feature model created. Shared feature ligand-based models were created based on the ligands from the corresponding PDB entries. Out of the automatically generated models, the model with highest score was selected. Automatically generated pharmacophore models usually profit from manual refinement and optimization by altering feature sizes, removing selected features, setting some features optional and adapting or removing exclusion volumes [3]. Therefore, every generated pharmacophore model was further refined by manual adaptation to enhance its sensitivity and specificity.

Dataset of GCPII ligands

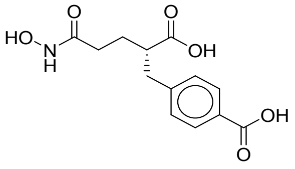

5D29

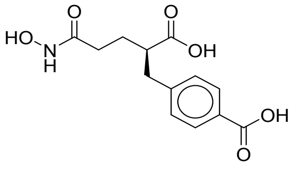

5ELY

Figure S1. Co-crystallized GCPII ligands binding S1 site subset.

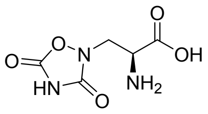

2JBK

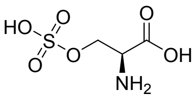

2PVV

Figure S2. Co-crystallized GCPII ligands binding S1’ site subset.

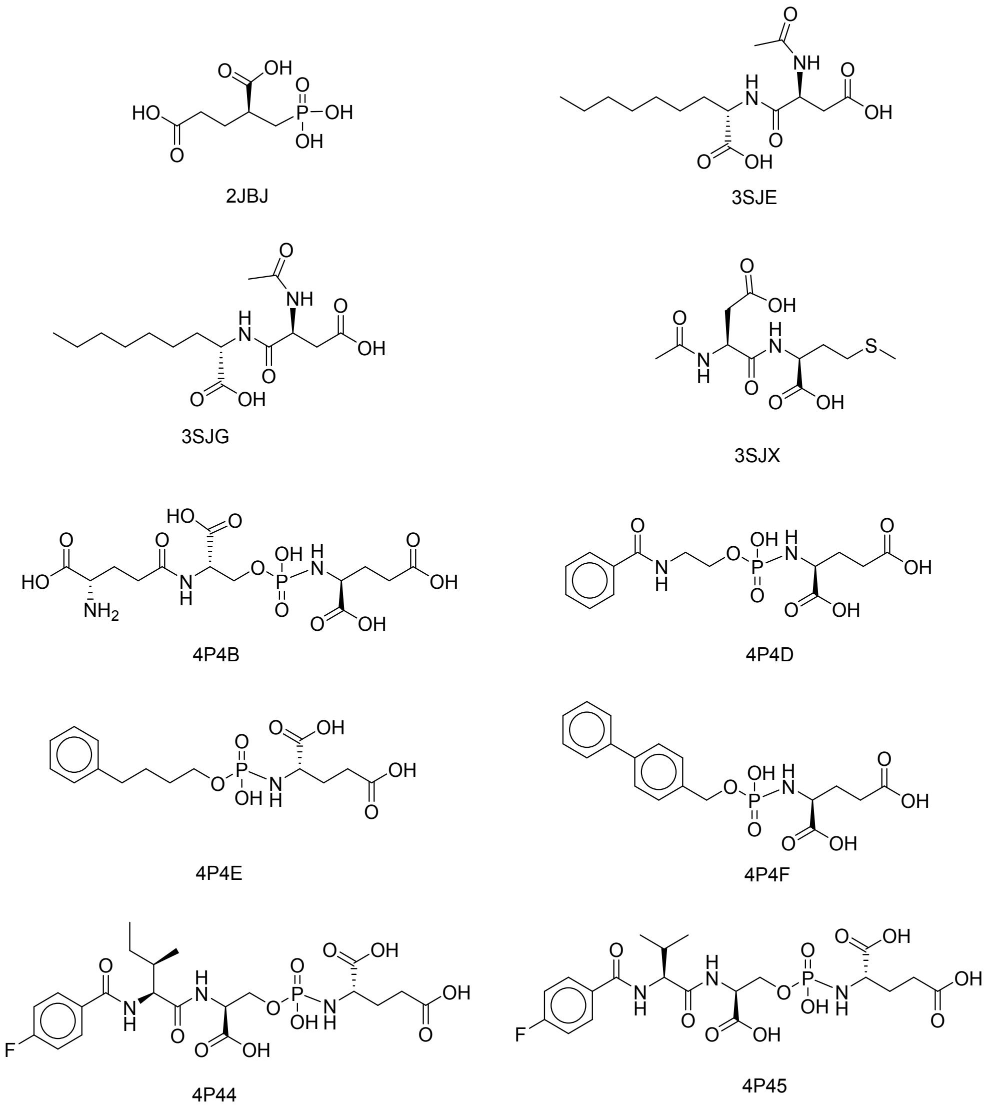

Figure S3. Smaller co-crystallized GCPII ligands binding S1 & S1’ site subset.

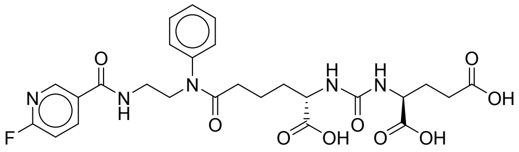

6HKZ

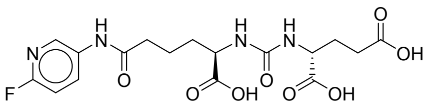

6HKJ

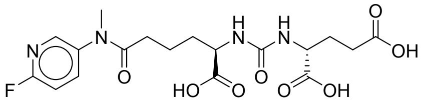

6H7Z

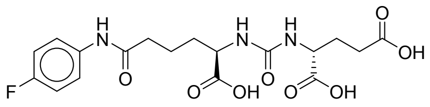

6H7Y

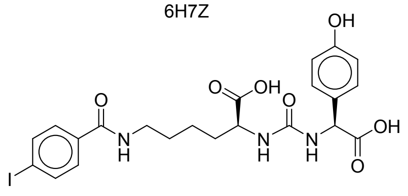

4OC5

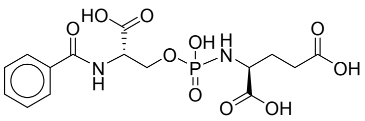

4P4I

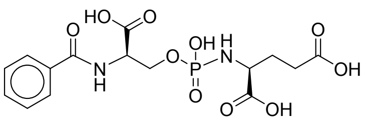

4P4J

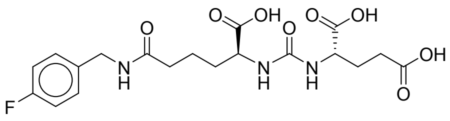

5OF0

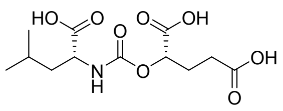

6ETY

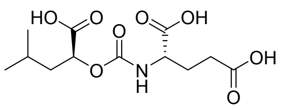

6EZ9

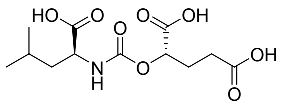

6F5L

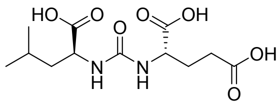

6FE5

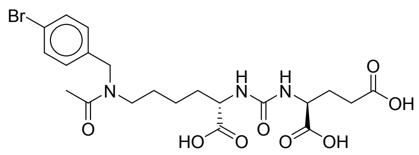

4NGM

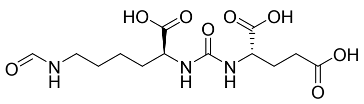

4NGN

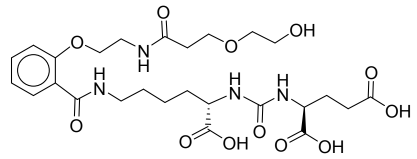

4NGP

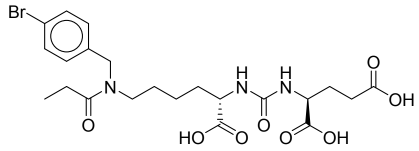

4NGQ

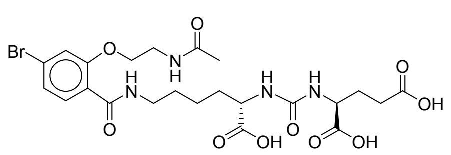

4NGR

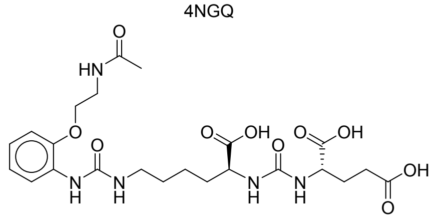

4NGS

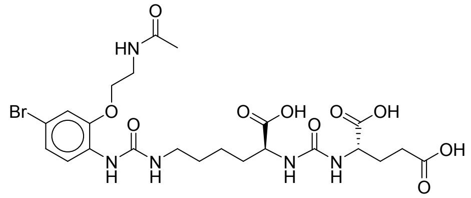

4NGT

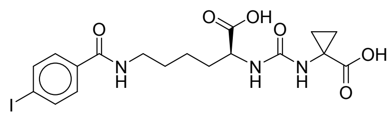

4OC0

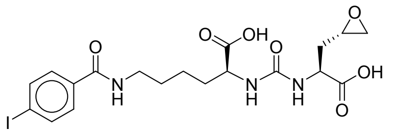

4OC1

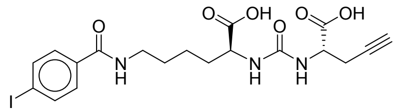

4OC2

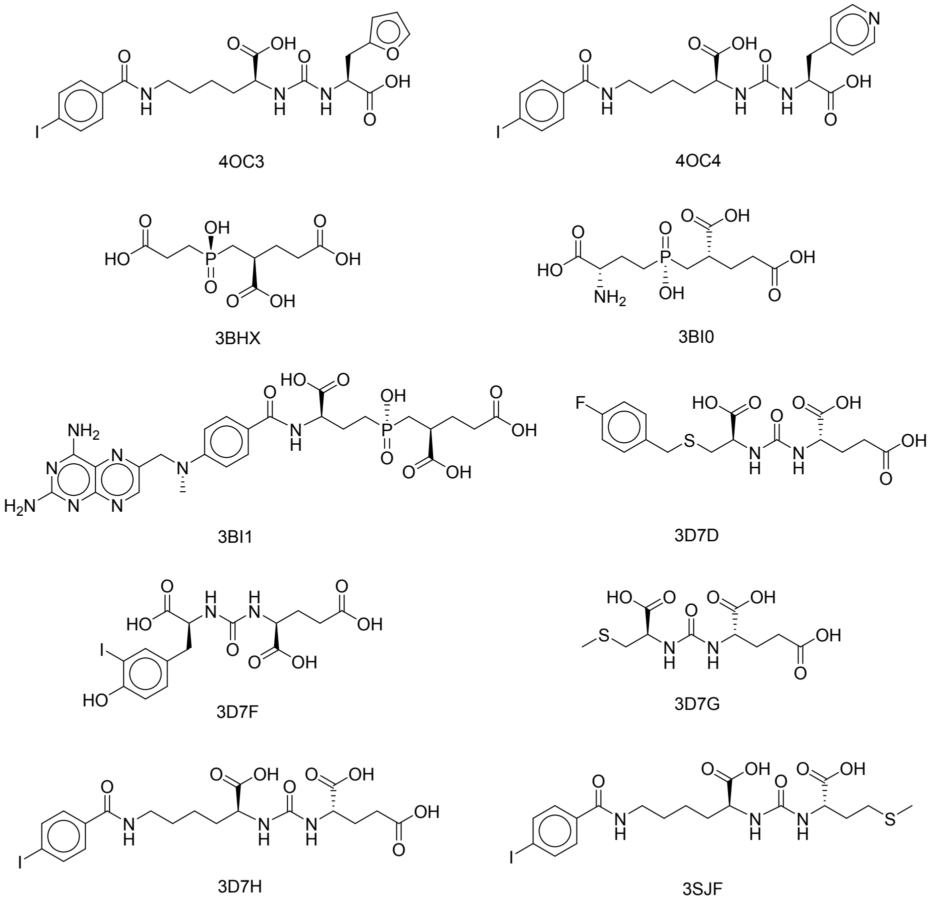

Figure S4. Larger co-crystallized GCPII inhibitors binding S1-S1'-site subset.

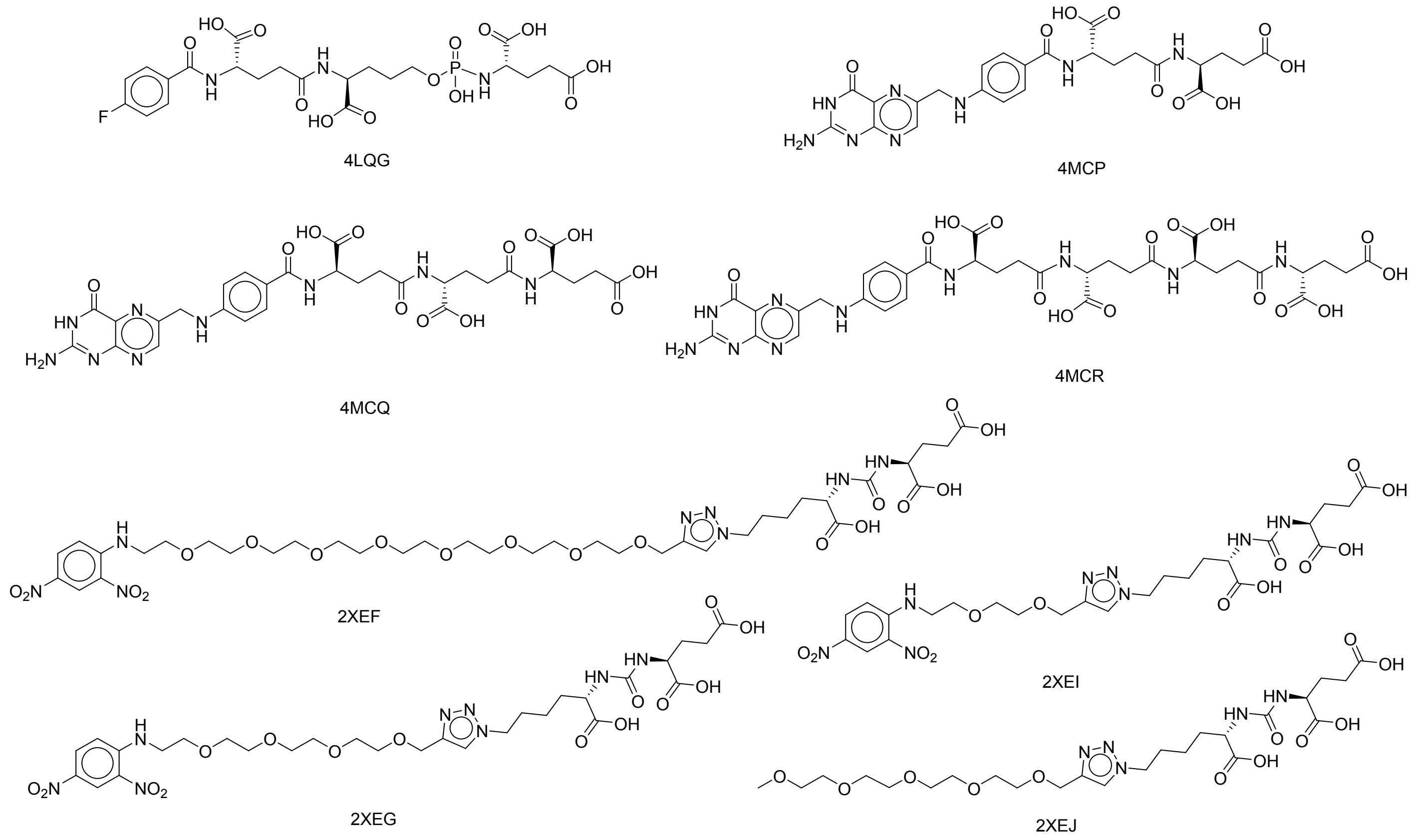

Figure S5. Co-crystallized GCPII inhibitors binding S1, S1’ and ABS site subset.

2. Description and optimization of pharmacophore models 1-8

Model 1

Model 1 was generated based on two enantiomeric inhibitors (JHU241 and JHU242) binding to the S1 site (PDB 5D29 and 5ELY) [4] (Fig. S6-B). Models for both structures were generated and combined to a shared feature pharmacophore model using the coordinates of 5ELY [4]. After the optimization process (Fig. S7), the model consisted of eight features and nine Xvols. The S1 site is located deep inside the binding pocket next to the catalytic Zn2+ ions. Key features of this model are therefore the zinc-binding and the NI feature. The binding group is stabilized by a HBD feature with Asp387 and a HBA with Tyr552, one of the amino acids delineating the S1 site. A hydrophobic contact, the second NI position interacting with Gly534 and two HBA features with Tyr234 and Arg534 (also a part of the S1), completed the spatial arrangement (Fig. 3A). This model was highly restrictive (Ef = 1341.5, maximum value) and only found the two compounds in the dataset.

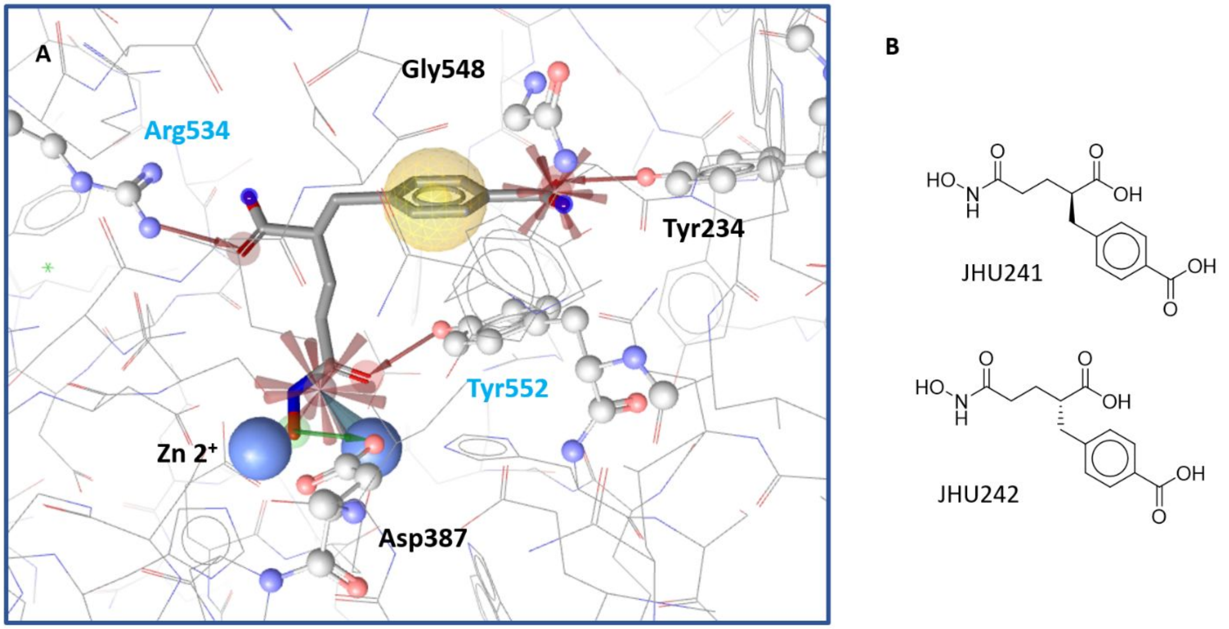

Figure S6. A) Pharmacophore model 1 with compound JHU242, based on the PDB entry 5ELY [4]. Blue spheres represent the zinc ions coordinating the ligand hydroxamate moiety. Red stars represent NI pharmacophore features, red arrows HBAs, green arrow HBDs, and the yellow sphere marks a HC B) Structures of inhibitors of the S1 subset.

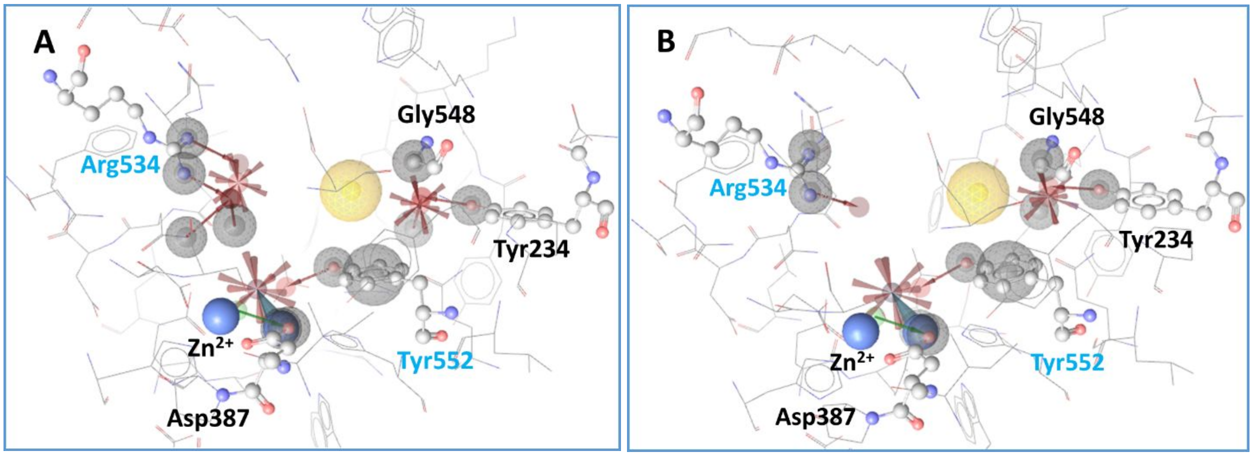

Figure S7. A) automatically generated structure-based pharmacophore model 1 (based on S1 site subset ligands shown in figure S1) B) the optimized model 1.

The originally generated model 1 consisted of 13 features (3 NIs, 7 HBAs, 1 HBD, 1 zinc binding and 1 HC) and 11 Xvols. The model optimization led to removal of the NI feature interacting with Arg534 and 4 HBA features as well (1 interacting with Arg534, 1 with Asn519 and 2 with water molecules). Moreover, 2 Xvols were removed during the model optimization (one placed on water molecule and one on nitrogen of Asn519). This resulted in a model depicted on Figure S7-B. Model 1 consists of 8 features (2 NIs, the first coordinating the zinc atoms and the second is placed next to Tyr234, 3 HBAs hydrogen bonding with Arg534, Tyr234 and Tyr552, 1 HBD interacting with Asp387, one HC and 1 zinc binding feature) and 9 Xvols.

Model 2

Model 2 (Fig S8) was generated based on three inhibitors, namely (2S)-2-[(N-acetyl-L-alpha-aspartyl)amino]nonanoic acid (pdb entry 3SJE [5]), (2S)-2-[(N-acetyl-L-alpha-aspartyl)amino]octanoic acid (3SJG [5]), and N-acetyl-aspartyl-methionine (3SJX [5]). The three automatically generated structure-based models were combined [5]. The resulting shared feature model was optimized to enrich active compounds over decoys. The final model consisted of nine features and 16 Xvols. Two zinc binding features anchored the molecule to the catalytic center and a feature complex consisting of an NI and two HBA features connected the molecule to the arginine patch (Arg534, Arg536). In the S1' site the pharmacophore contained another NI, interacting with Arg210 and two HBAs, one engaging Arg210 and the second a conserved water molecule bound to Asn257, Trp381, and Glu425. The model correctly identified ten out of twelve actives for this binding site and 40 decoys. The enrichment metrics are shown in Table 1. This model was less restrictive than model 1 and covered mainly smaller molecular entities.

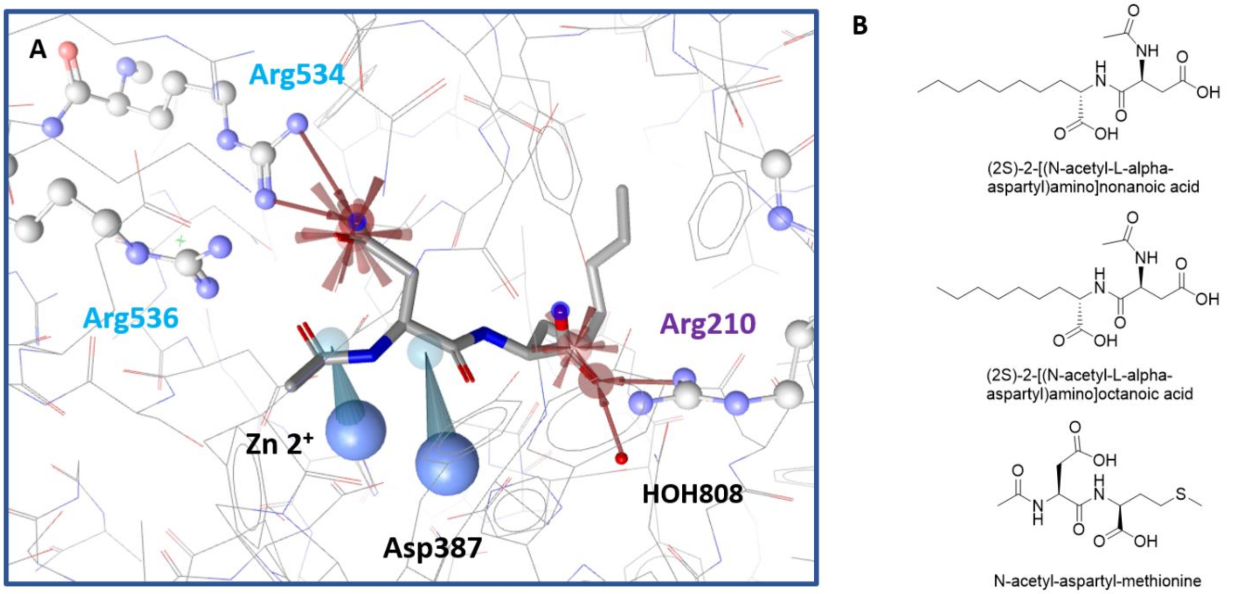

Figure S8. A) Model 2 with (2S)-2-[(N-acetyl-L-alpha-aspartyl)amino]octanoic acid in the binding pocket of PDB entry 3SJG [5]. The model consisted of two zinc-binding features, two NI features, and 4 HBAs. B) The model was based on three ligands co-crystallized with GCPII (3SJG, 3SJX, and 3SJE [5]).

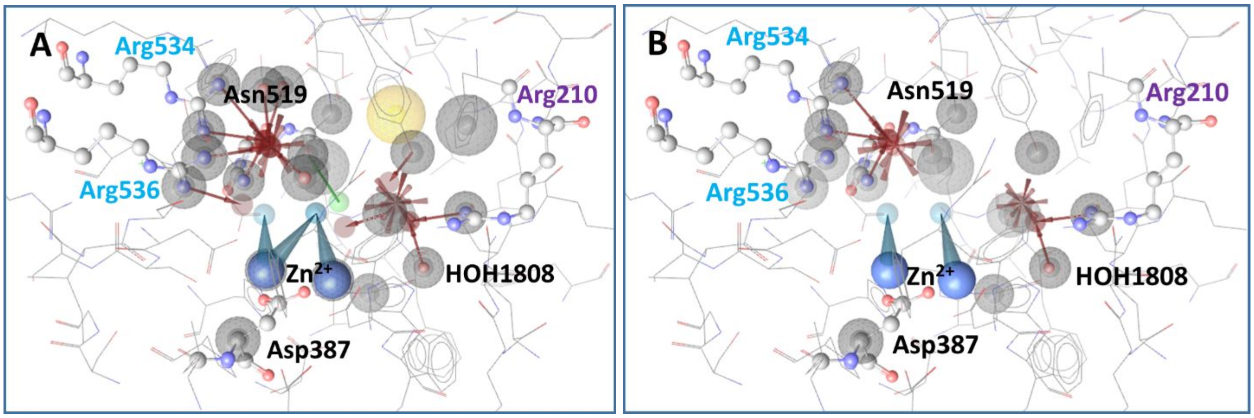

Figure S9. A) automatically generated structure-based pharmacophore model 2 (based on small S1-S1' site subset ligands shown in figure S3) B) the optimized model 2.

The originally generated model 2 consisted of 17 features (2 NIs, 10 HBAs, 1 HBD, 3 zinc binding and 1 HC) and 21 Xvols. The following features were removed during the model optimization process. 4 HBAs (1 forming interaction with Tyr700, 1 with Tyr552 and 2 with Arg536), the HBD with Asn519, 1 zinc binding feature, and the HC feature. One HBA feature with water molecule HOH1964 was removed, while HBA feature with HOH1808 was kept since this feature significantly increased model performance. Moreover, 4 Xvols were removed as well. The final optimized model 2 consists of 9 features (2 NIs, 1 interacting with Arg210 while the second with Arg534 and Arg536, 5 HBAs - 2 with Arg534, 1 with Asn519, 1 with Arg210 and the last one with HOH1808, 2 zinc binding features,) and 16 Xvols.

Model 3

Structure-based pharmacophore model 3 (Figure S10) was built based on two GCPII ligands, L-serine-O-sulfate (PDB entry 2PVV) [6] and quisqualic acid (PDB entry 2JBK) [7]. Two structure-based models were built on both complexes, overlaid (reference: 2JBK) and a shared feature pharmacophore model was generated. Such automatically generated model consisted of 8 features (1 NI, 1 PI, 3 HBAs, and 3 HBDs) and 8 Xvols. After removing 1 HBA and 2 HBDs interacting with water molecules, the model was optimized to map these two ligands only. The resulting model 3 consists of 5 features (1 NI interacting with Arg210, 1 PI interacting with Glu424, 2 HBAs interacting with Arg210 and Tyr700, and 1 HBD interacting with Glu424) and is depicted on Figure S8-B.

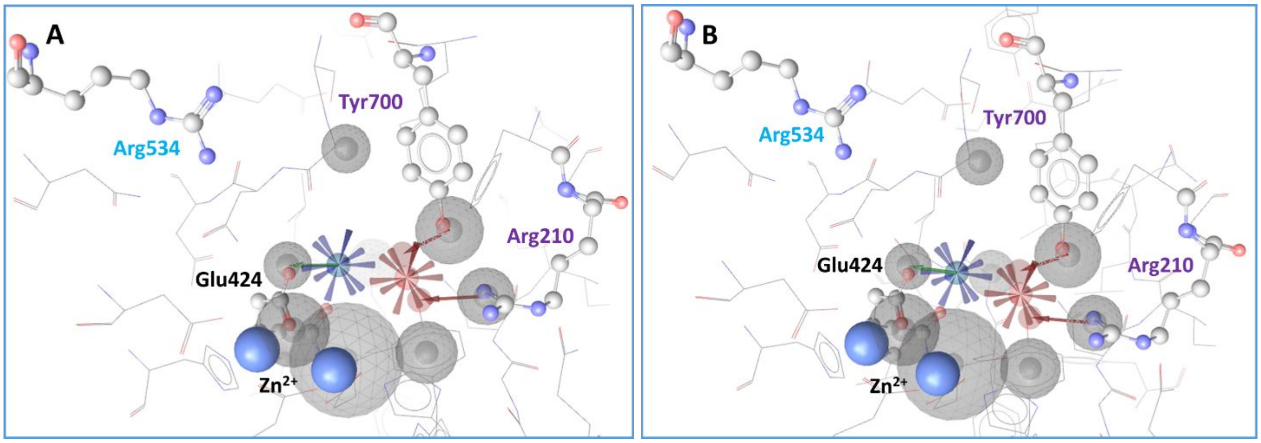

Figure S10. A) Shared feature structure-based pharmacophore model 3 representing two inhibitors of the S1'-subset based on PDB entries 2JBK and 2PVV (two ligands of the S1'-subset shown in figure S2). B) The optimized model 3.

Model 4

The description and depiction of the model is shown in the main manuscript.

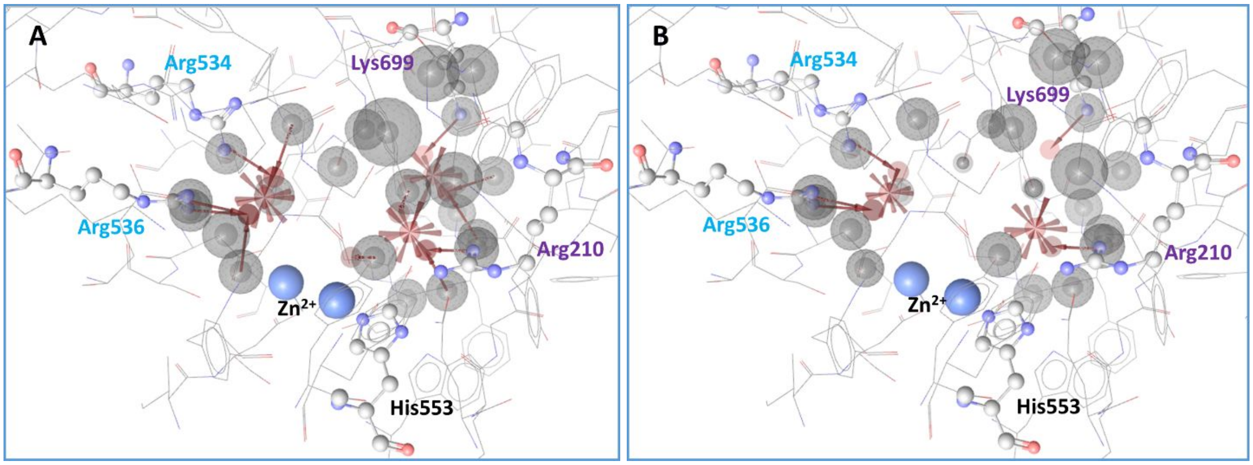

Figure S11. A) automatically generated structure-based pharmacophore model 4 for the GCPII inhibitors of the S1-S1' subset (figure S4) and B) the optimized model 4.

The automatically generated model 4 consisted of 15 features (12 HBAs and 3 NIs) and 22 Xvols. The NI feature interacting with Lys699 was removed in the optimization process. 7 HBAs were removed as well, 1 interacting with Asn257, 1 with Tyr552, 1 with Tyr700 and 4 with water molecules. The optimized model 4 consists of 7 features (2 NIs, one interacting with Arg534 and Arg536 and the second with Arg210 and His553, 5 HBAs, 1 forming an interaction with Arg534, 2 with Arg536, 1 with Lys699 and 1 with Arg210) and 19 Xvols.

Models 5 and 6

A shared-feature ligand-based model based on the same compounds as the structure-based model 4 (Fig. 3B) was created to allow for more flexibility in the hydrogen bonding angles. Two models resulted from the optimization process: Model 5 (Fig. S12-A) contained 37 Xvols, two NIs and four HBA features that were located at similar locations as in model 4 but were not constrained by specific binding angles. An additional HBD feature was introduced that interacted with Glu424 or Gly518 residues of the enzyme. Model 6 was very similar to the model 5, but lacked one HBA feature and contained only 25 Xvols. Model 6 (Fig. S12-B) was slightly less restrictive than model 5 and allowed us to find all 32 actives in the S1&S1'large dataset in combination with models 5 and model 4. The enrichment metrics of both models are shown in Table 1.

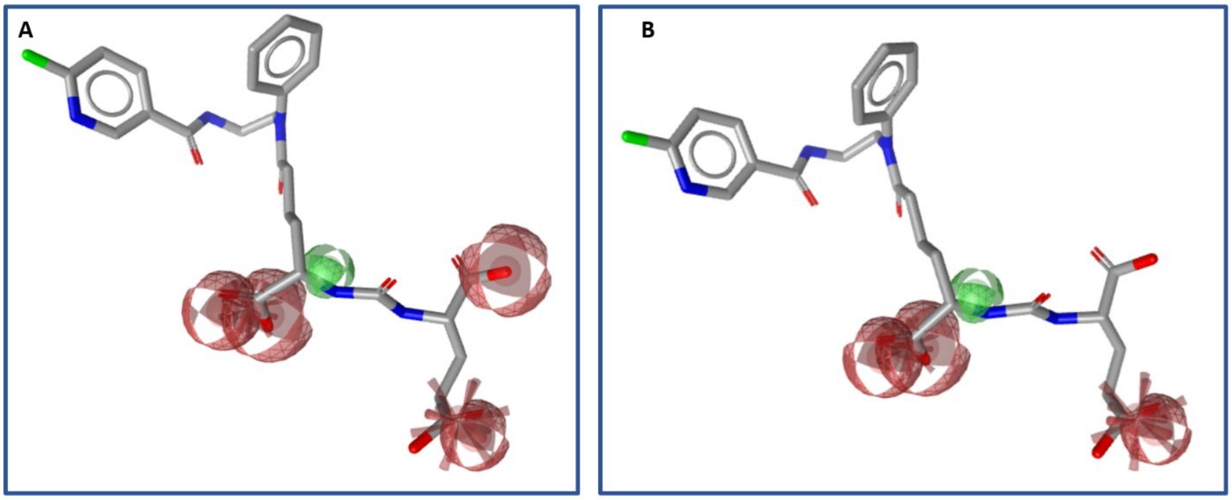

Figure S12. A) Model 5: Shared-feature ligand-based pharmacophore model for the S1&S1'-subset with ligand RNA 2-49-1. Red and green spheres mark HBA and HBD features respectively. B) Model 6: Shared feature ligand based pharmacophore model for the S1&S1'-subset with ligand RNA 2-49-1.

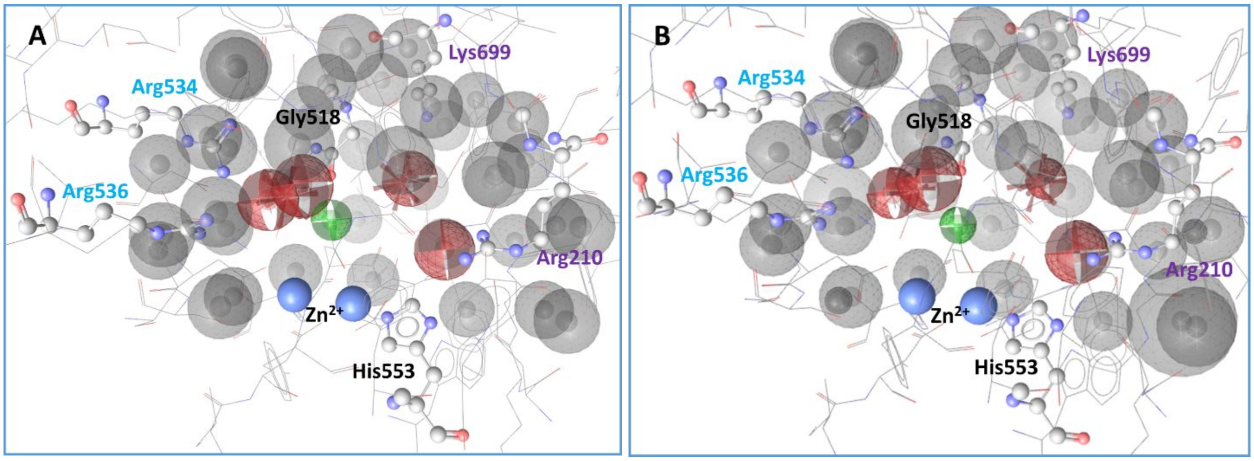

Figure S13. A) automatically generated ligand-based pharmacophore model 5 for the GCPII inhibitors of the S1-S1' subset (figure S4) B) the optimized model 5.

The automatically generated model that served as a basis for models 5 and 6 consisted of 7 features (4 HBAs, 2 NIs, and 1 HBD) and 37 Xvols. The amount of model features and tolerances were subsequently optimized and the best performance achieved when the HBA feature interacting with Arg210 and His553 was set to optional. Since optional feature increases computational costs of the screening process, we decided to split this model into two, model 5 where this feature was obligatory (Figure S10-B) and model 7 where this feature was removed (Figure S14-B). The optimized model 5 consists of the same 7 features and 37 Xvols.

Overlay of this model with model 4 indicates that one NI feature of this ligand-based model would possibly interact with Arg534 and Arg536 of GCPII while the second NI with Lys699. The NI feature interacting with Arg210 and His553 in model 4 was not generated in this ligand-based model. The HBD would interact with Gly518. The HBA present in model 5 but not in model 6 would potentially interact with Arg210 and His553. The second HBA feature with Lys699 while the third and fourth HBA features, being close to each other interact with Arg534, Arg536 and Asn519.

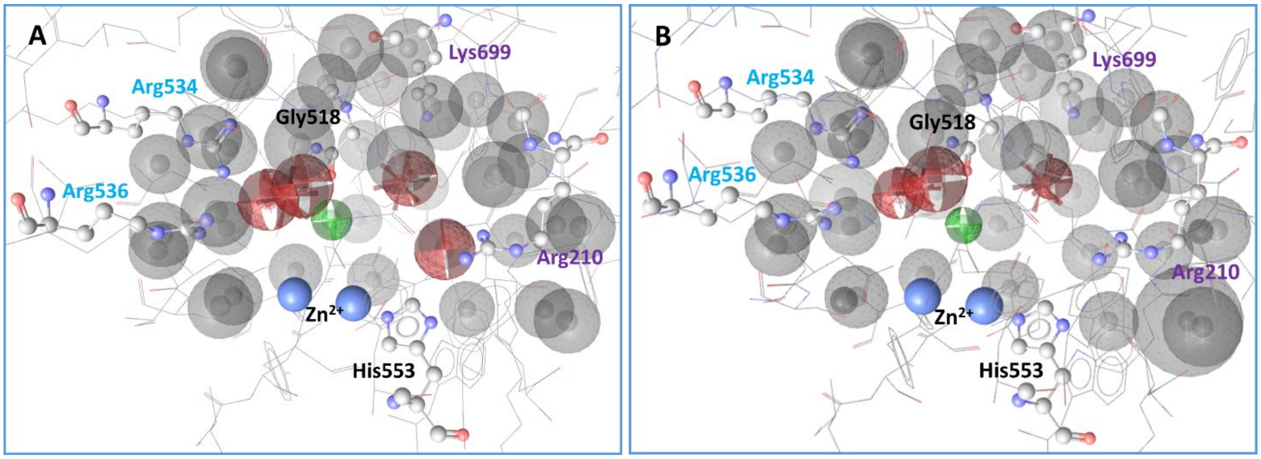

Figure S14. A) automatically generated ligand-based pharmacophore model 6 for the GCPII inhibitors of the S1-S1' subset B) the optimized model 7.

Model 7

The subset spanning S1, S1'& ABS encompassed eight very large inhibitors (Fig. S5). Structure-based model 7 was based on the co-crystallized inhibitor ARM-P4 and consisted in total of 9 features and 29 Xvols (Fig. S15-A). Two of the NI features interacted with Arg210 and Lys699 of the S1' pocket, while the third NI bound to Arg534 of the S1 pocket. Furthermore, the six HBA features interacted with residues of either specificity pocket. The Xvol coat allowed for the molecule to fill all three pockets of the binding site. The model found all 8 inhibitors of the training set and 14 decoys leading to an Ef = 122.2 (Table 1).

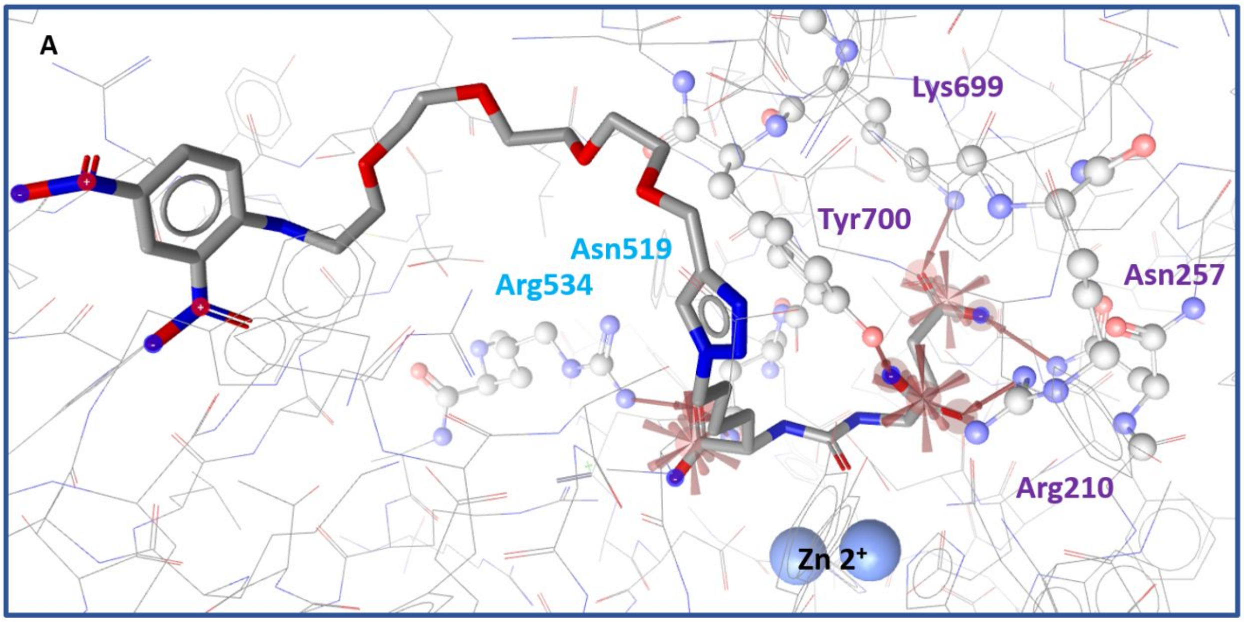

B

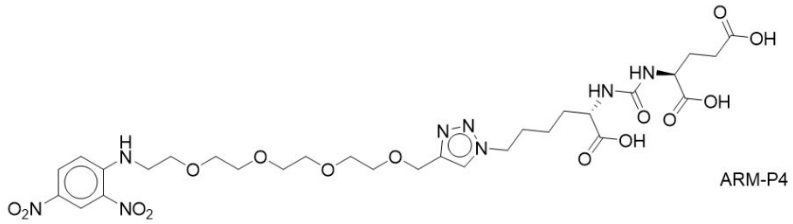

Figure S15. A) Model 7: Structure-based pharmacophore model for the S1-S1'& ABS-subset created based on PDB entry 2XEG [8] visualized with the ligand ARM-P4. B) Antibody recruiting GCPII inhibitor ARM-P4 used for model generation.

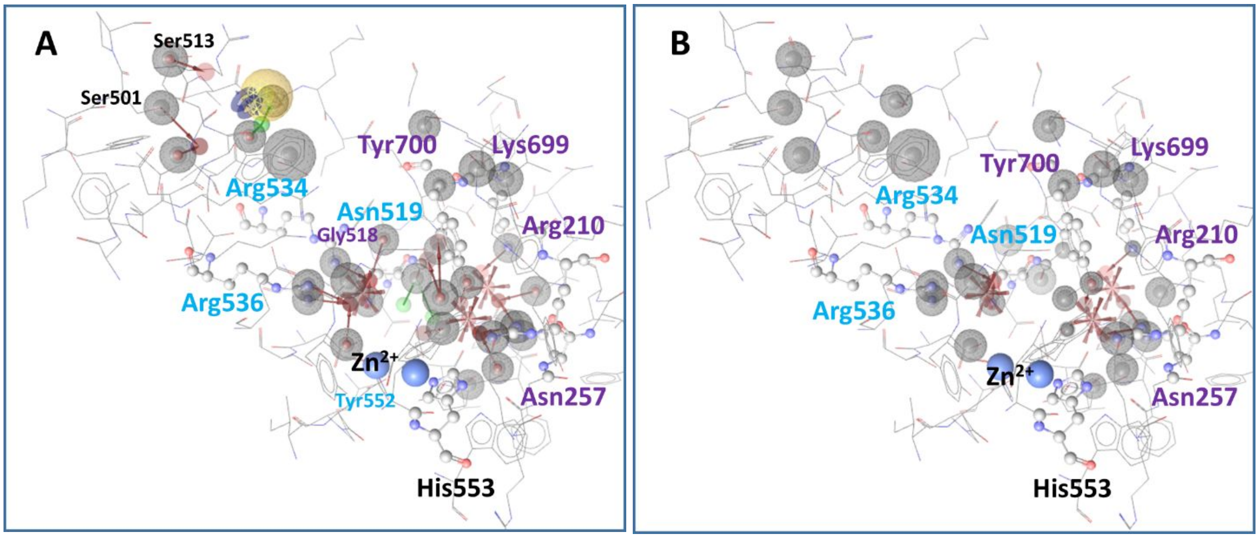

Figure S16. A) automatically generated structure-based pharmacophore model 7 for the GCPII inhibitors of the S1-S1' subset (figure S5) based on PDB ID 2XEG and B) the optimized model 7.

The automatically generated model 7 consisted of 28 features (3 NIs, 19 HBAs, 4 HBDs, 1HC and 1 aromatic) and 30 Xvols. 8 HBAs interacting with water molecules were removed during model optimization. Similarly, 5 more HBAs were removed, 1 interacting with Tyr552, 2 with Arg536, 1 with Ser501 and 1 with Arg210. The 2 HBDs interacting with Gly518 and 1 with Ser513 were removed as well as HBD interacting with water molecule. The aromatic feature as well as the hydrophobic contact were removed as well.

The optimized model 7 consists of 9 features (3 NIs and 6 HBAs) and 29 Xvols (Fig S15). One of the NI features interacts with Arg534 and Arg536, the other with Arg210 and His553 and the last one with Lys699. 1 HBA forms an interaction with Tyr700, 1 with Arg210, 1 with Lys699 and 1 with Asn257, 1 with Arg534 and 1 with Asn519.

Model 8

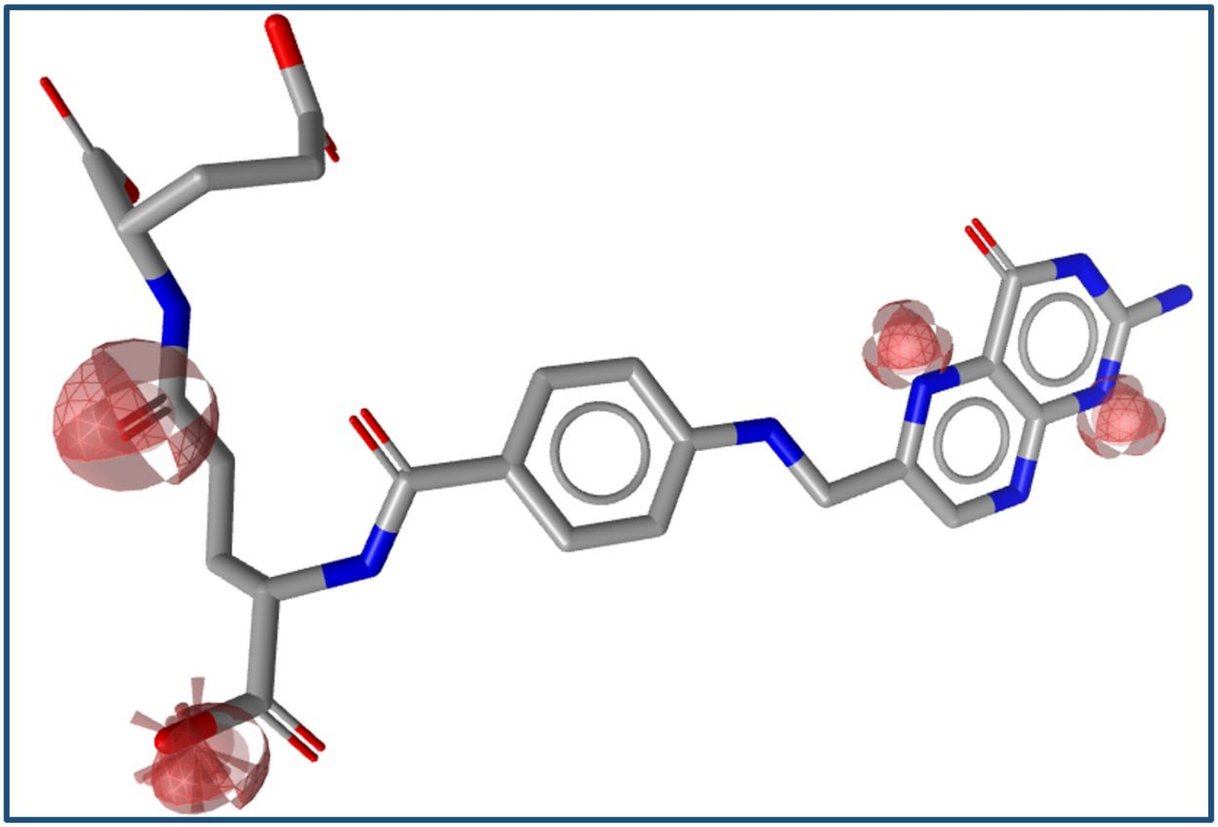

Figure S17. A) Shared-feature-ligand-based pharmacophore model 8 for the S1-S1'&ABS-subset created based on all eight inhibitors co-crystallized in this binding site shown with folyl-gamma-L-glutamic acid.

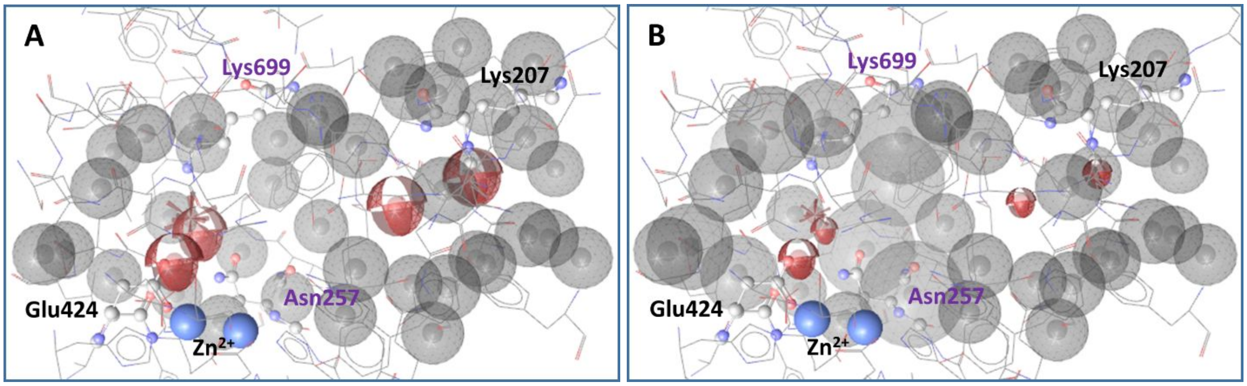

Figure S18. A) automatically generated ligand-based pharmacophore model 8 for the GCPII inhibitors of the S1-S1' subset (figure S5) and B) the optimized model 8.

The automatically generated model 8 consists of 5 features (4 HBAs, 1 NI) and 39 Xvols. Optimization of this model consisted mostly of increasing the size of Xvols and decreasing the tolerances of the HBA and NI features. The optimized model 8 consists of the same 5 features and 39 Xvols.

Overlay of this model with model 7 indicates that NI of this ligand-based model would possibly interact with Lys699 of GCPII. 1 HBA feature shows a potential to interact with Glu424, 1 with Asn257, 1 with the backbone nitrogen of Lys207 while the interaction partner of the last HBA remains unknown.

Table S1. Enrichment metrics of the pharmacophore models 1,2 and 4-8. All 54 GCPII active compounds regardless of their binding mode were considered when calculating the parameters. The database of decoys was composed of 2681 compounds (TP - true positive; FP - false positive; TN - true negative; FN – false negative; YoA – yield of actives; EF – enrichment factor; AUC – area under the curve).

Model	1	2	4	5	6	7	8	all
**TP**	2	32	36	25	41	36	8	54
**FP**	0	40	21	10	36	14	4	90
**TN**	2681	2641	2660	2671	2645	2667	2677	2591
**FN**	52	22	18	29	13	18	46	0
**# of actives in the database**	54	54	54	54	54	54	54	54
**decoy hitrate [%]**	0.00	1.49	0.78	0.37	1.34	0.52	0.15	3.36
**accuracy**	0.98	0.98	0.99	0.99	0.98	0.99	0.98	0.97
**YoA**	1.00	0.44	0.63	0.71	0.53	0.72	0.67	0.38
**EF**	50.65	22.51	31.99	36.18	26.97	36.47	33.77	18.99
**EF_{max}**	50.65	50.65	50.65	50.65	50.65	50.65	50.65	50.65
**EF/EF_{max}**	1.00	0.44	0.63	0.71	0.53	0.72	0.67	0.38
**sensitivity**	0.04	0.59	0.67	0.46	0.76	0.67	0.15	1.00
**specificity**	1.00	0.99	0.99	1.00	0.99	0.99	1.00	0.97
**AUC**	0.50	0.71	0.83	0.73	0.87	0.83	0.57	0.99

Table S2. Overview of the tested virtual hits. Percent inhibition of GCPII activity of the 50 experimentally tested virtual hits. The pharmacophore model that identified the hits is also indicated. Compounds were tested at concentration of 20 μM with enzymatic activity assay using highly purified human recombinant GCPII and fluorescently labeled Glu-Glu dipeptide. SD – standard deviation. Compounds with more than 50% inhibition are marked in green, compounds with more than 25% inhibition are marked in yellow.

Cmpd ID	SPECS ID	Inh. [%]	SD	model	Cmpd ID	SPECS ID	Inh. [%]	SD	model
**1**	**AG-690/36543021**	**0**	0	2, 5 & 6	**26**	**AE-848/37236067**	18	2	2
**2**	**AG-205/09230056**	**0**	0	2, 5 & 6	**27**	**AT-051/43422486**	18	1	2
**3**	**AE-848/37236064**	**11**	2	2, 5 & 6	**28**	**AN-465/43098644**	29	3	2
**4**	**AQ-099/42181991**	**2**	2	2 & 4	**29**	**AE-848/32324022**	0	1	8
**5**	**AP-853/43445396**	**3**	3	2 & 4	**30**	**AN-329/40942995**	0	0	8
**6**	**AP-263/40778381**	**4**	2	2 & 6	**31**	**AN-465/43411236**	8	3	6
**7**	**AE-473/30080064**	**12**	4	2 & 6	**32**	**AP-866/40876198**	1	1	6

**8**	**AP-263/43479126**	**1**	1	2 & 6	**33**	**AG-690/36951091**	0	0	6
**9**	**AK-918/12902970**	**0**	0	2 & 6	**34**	**AU-059/43517551**	2	2	6
**10**	**AK-918/37196003**	**0**	0	2 & 6	**35**	**AG-205/07465023**	1	2	6
**11**	**AK-918/11641024**	**0**	0	2 & 6	**36**	**AP-263/41686904**	3	3	6
**12**	**AG-690/12245693**	**0**	0	2 & 6	**37**	**AP-263/12245698**	1	1	6
**13**	**AP-263/40917807**	**0**	0	2 & 6	**38**	**AF-399/15335468**	0	0	6
**14**	**AN-465/43411093**	**0**	0	2 & 6	**39**	**AP-845/41686908**	10	4	6
**15**	**AG-690/12242412**	**13**	3	2 & 6	**40**	**AP-263/41686907**	4	4	6
**16**	**AP-263/14677255**	**0**	0	2 & 6	**41**	**AP-263/40971777**	0	0	6
**17**	**AN-967/15490027**	**88**	12	4 & 7	**42**	**AQ-390/40847774**	1	1	4
**18**	**AL-291/37110008**	**61**	6	4 & 7	**43**	**AG-690/32524014**	1	1	4
**19**	**AQ-390/42869246**	**0**	0	5 & 6	**44**	**AO-548/41044785**	0	0	4
**20**	**AQ-086/43383919**	**8**	5	2	**45**	**AP-970/42983384**	1	1	4
**21**	**AR-422/43115151**	**1**	1	2	**46**	**AN-652/13064001**	99	2	4
**22**	**AE-848/37236070**	**38**	2	2	**47**	**AP-406/42800686**	24	3	4
**23**	**AI-204/31703001**	**11**	4	2	**48**	**AN-652/12812004**	16	5	4
**24**	**AE-562/43282615**	**33**	5	2	**49**	**AU-059/43515307**	15	6	4
**25**	**AH-262/02650039**	**0**	0	2	**50**	**AG-205/33006036**	1	2	4

3. *Selection of virtual SPECS hits for experimental activity testing*

The 82 SPECS hits that mapped our models were narrowed down to 50 compounds for which experimental inhibitory activity against GCPII was evaluated. To this end, following steps were used to filter SPECS hits.

1. Compounds resembling natural GCPII substrate were excluded (all compounds containing glutamate with an amide bond on N terminal).
2. SciFinder similarity search was performed for all virtual hits. If the hit was already mentioned/tested against GCPII, it was discarded.
3. Compounds to test were selected with emphasis on their structural diversity that was assessed visually.
4. Since consensus hits (virtual hits in more than one model) are supposed to have a higher probability of being true positives than virtual hits of a single model, we aimed to experimentally test all consensus hits except if they failed criterion 1, 2, or 3.
5. Compounds for experimental activity evaluation were selected aiming to represent every pharmacophore model by the same amount of virtual hits (where possible). If the model mapped too little SPECS compounds, all those satisfying the previous two criteria were tested.

Three of the four consensus hits identified concomitantly by models 2, 5 and 6 were experimentally tested on their GCPII inhibitory activity. Compound AN-329/40945305 (we refer the reader to www.specs.net) was not selected for experimental testing due to its striking similarity to 1 (Figure S13) and due to SciFinder (scifinder.cas.org) similarity search uncovered two compounds that were already tested against GCPII.

From the 19 compounds that were mapped by two of our pharmacophore models, 16 were selected for experimental activity testing (Figure S13). The three excluded compounds were all identified by both models 2 and 7. Compound AE-562/40806920 was removed since its exact search in SciFinder returns more than 12K results rendering this compound highly likely to be already tested on many targets. Compound AG-690/12245692 was excluded due to its high similarity to two other double consensus hits, 12 (Figure S13) and 13 (Figure S13). Compound AN-584/43503783 was not experimentally tested because of its small molecular weight of 171.152.

All single hits of models that found less than 10 compounds (models 5, 7 and 8, Table S3) were selected for testing. Model 4 identified 15 compounds. They were all experimentally tested except for compound AG-690-33064013 due to its striking similarity to compound 43 and to 50 (AG-205/33006036)

Model 2 and model 6 mapped the most SPECS hits of all the models generated, 25 and 26, respectively. Selection of SPECS hits for experimental testing of these models was two-step process. First, hits were ordered according to their descending pharmacophore fit score. Second, the highest-ranking compounds were selected for testing. However, if the compound was visually similar to compounds already selected for testing from other models, or to higher ranked compounds of these models, it was discarded. Summary of the compounds tested from each model is shown Table S3 and Figure S13.

Table S3. Number of compounds selected for experimental inhibitory activity testing. 50 compounds were tested in total, of those 19 consensus hits and 31 individual hits.

model	total	consensus hits	individual hits
**2**	25	16	9
**4**	14	5	9
**5**	3	3	0
**6**	26	15	11
**7**	2	2	0
**8**	2	0	2
**total**	50	19	31

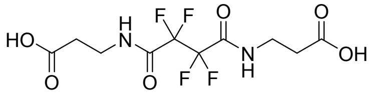

compound 1
AG-690/36543021

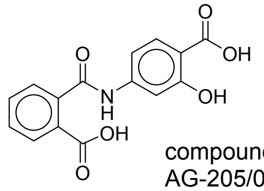

compound 2
AG-205/09230056

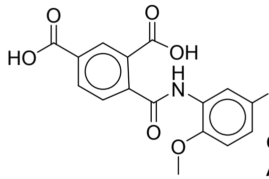

compound 13
AP-263/40917807

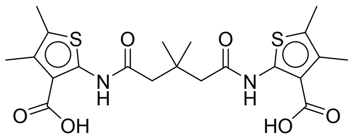

compound 3
AE-848/37236064

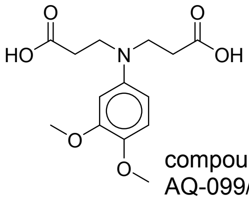

compound 4
AQ-099/42181991

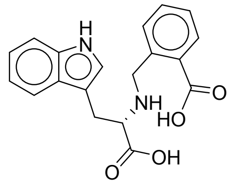

compound 14
AN-465/43411093

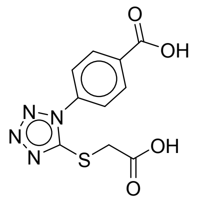

compound 5
AP-853/43445396

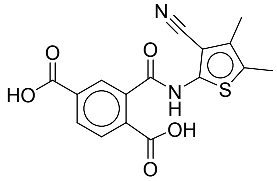

compound 6
AP-263/40778381

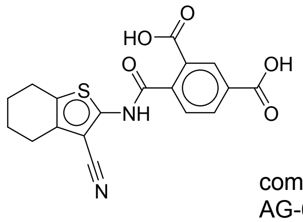

compound 15
AG-690/12242412

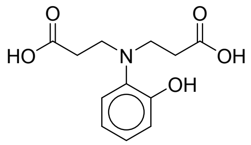

compound 7
AE-473/30080064

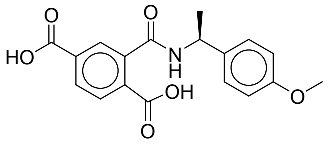

compound 8
AP-263/43479126

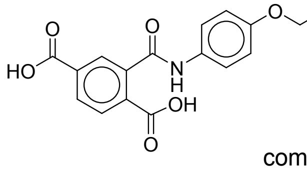

compound 16
AP-263/14677255

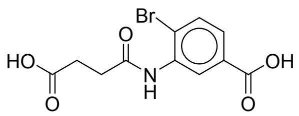

compound 9
AK-918/12902970

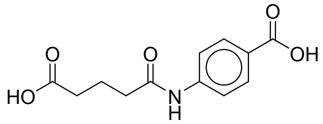

compound 10
AK-918/37196003

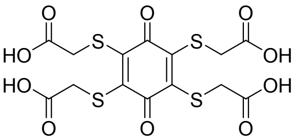

compound 17
AN-967/15490027

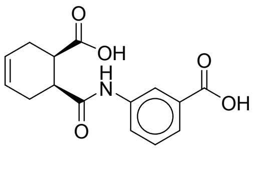

compound 11
AK-918/11641024

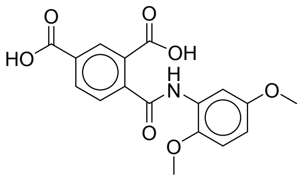

compound 12
AG-690/12245693

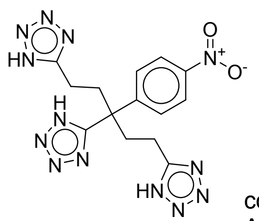

compound 18
AL-291/37110008

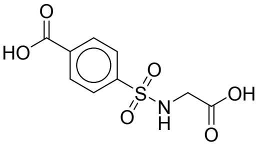

compound 19
AQ-390/42869246

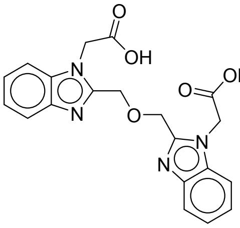

compound 20
AQ-086/43383919

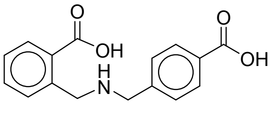

compound 31
AN-465/43411236

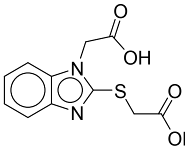

compound 21
AR-422/43115151

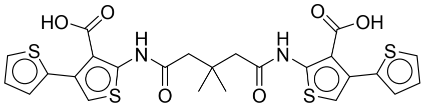

compound 22
AE-848/37236070

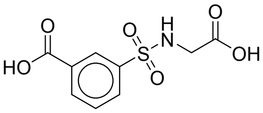

compound 32
AP-866/40876198

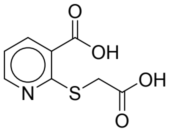

compound 23
AI-204/31703001

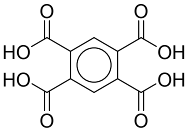

compound 24
AE-562/43282615

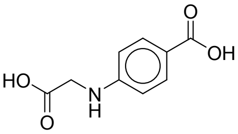

compound 33
AG-690/36951091

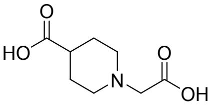

compound 25
AH-262/02650039

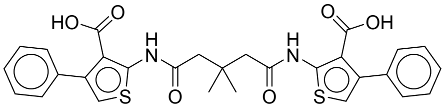

compound 26
AE-848/37236067

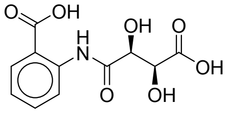

compound 34
AU-059/43517551

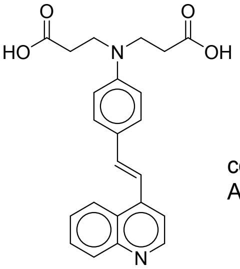

compound 27
AT-051/43422486

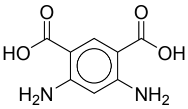

compound 28
AN-465/43098644

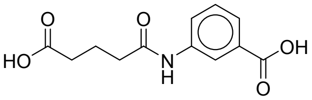

compound 35
AG-205/07465023

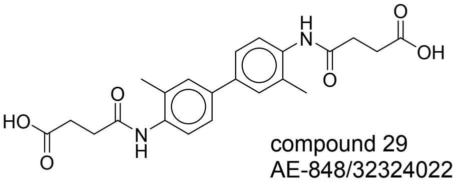

compound 29
AE-848/32324022

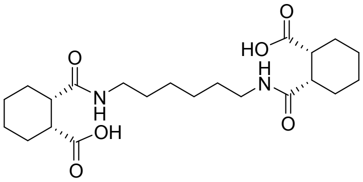

compound 30
AN-329/40942995

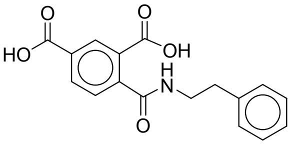

compound 36
AP-263/41686904

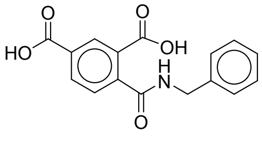

compound 37
AP-263/12245698

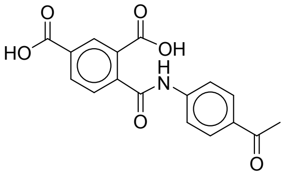

compound 38
AF-399/15335468

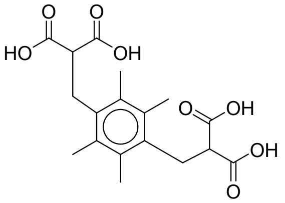

compound 47
AP-406/42800686

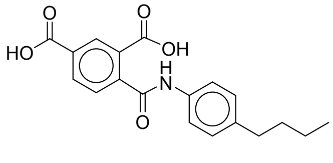

compound 39
AP-845/41686908

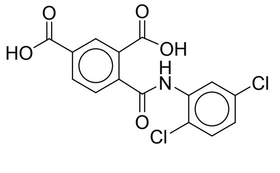

compound 40
AP-263/41686907

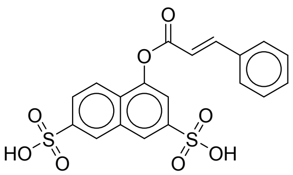

compound 48
AN-652/12812004

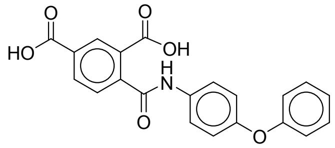

compound 41
AP-263/40971777

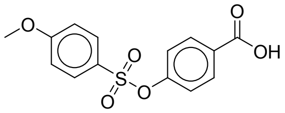

compound 42
AQ-390/40847774

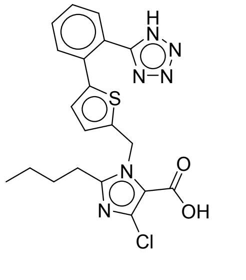

compound 49
AU-059/43515307

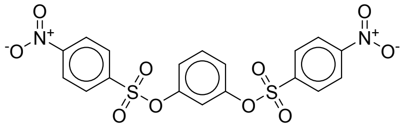

compound 43
AG-690/32524014

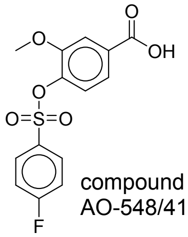

compound 44
AO-548/41044785

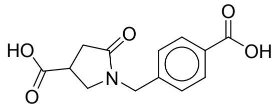

compound 45
AP-970/42983384

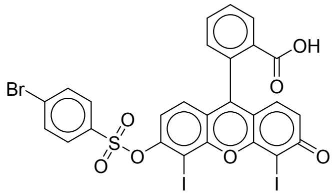

compound 46
AN-652/13064001

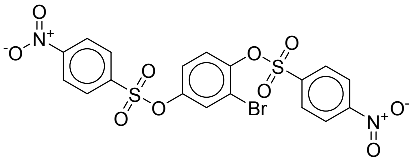

compound 50
AG-205/33006036

Figure S19. 50 compounds selected for experimental activity testing against GCPII. Compounds within the hitlist of a specific model or within the consensus hitlist of specific models are numbered according to their pharmacophore fit score in descending order. For consensus hitlists, pharmacophore fit score of the most restrictive model (based on EF) is taken into consideration.

Compounds 1-3: consensus hits of models 2, 5 and 6; compound 4 and 5: consensus hits of model 2 and model; compound 6 through 16: consensus hits of model 2 and model 6; compound 17 and 18: consensus hits of model 4 and model 7; compound 19 is consensus hit of model 4 and model 6; compound 20-28 are hits of model 2; compound 29-30 are hits of model 8; compound 31-41 are hits of model 6; compound 42-50 are hits of model 4

4. Generation of decoys dataset

To generate a set of decoys specific for GCPII, physico-chemical properties of all 54 GCPII active compounds (logP, molecular weight, number of hydrogen bond donating and accepting groups and number of rotatable bonds) were calculated using small molecules module in Discovery Studio [9]. Their statistical analysis is shown in Table S4. As clearly visible from the table, GCPII inhibitors are rather polar compounds mostly possessing many HBA and HBD features.

Table S4. Statistical physico-chemical characteristics of the 54 compounds in the active dataset (upper part) and the limits used to filter CHEMBL database [10] compounds (lower part) (HBA – hydrogen bond acceptor, HBD – hydrogen bond donor).

	**dataset of 54 active compounds**				
	AlogP [11]	molecular weight	HBA features	HBD features	rotatable bonds
average	0.02	468.07	9.52	5.65	13.56
minimum	-5.63	185.16	6	3	3
maximum	2.65	918.90	22	12	41
median	0.45	461.75	8	5	12
modus	0.88	305.28	8	5	12
	**GCPII-adapted Lipinski-like filter**				
minimum	-2.50	280	6	3	3
maximum	2.65	745	17	8	23

Only two compounds in the dataset of GCPII active compounds have molecular weight higher than 745 and three lower than 280. Only three compounds comprise more than 17 HBAs, only two more than 8 HBDs, only four more than 23 rotatable bonds (Table S4). Therefore, for the construction of decoys, only compounds with properties within the boundaries of our GCPII-adapted Lipinski-like filter (Table SI4) were selected.

The set of decoys was generated by stepwise filtering of more than 2 million bioactive molecules publicly available in the ChEMBL database [10]. Since many compounds in ChEMBL database contain experimental logP values that we wanted to retain, we first filtered out all compounds with missing logP value narrowing the database down to approximately 1.4 million compounds. In the second, our GCPII-adapted Lipinski-like filter reduced the ChEMBL database into 97 400 compounds that, in essence, have similar physico-chemical properties to GCPII active compounds. Application of in-house Pipeline Pilot protocol [12] clustered these compounds into 2700 clusters based on their Tanimoto similarity [13] using FCFP4 fingerprint [14,15]. One molecule from each cluster was selected for the decoys generating a decoy set of 2700 compounds.

5. PAINS analysis of hit compounds

Test compounds were not excluded based on PAINS filtering, we did however subject them to screening with a PAINS filter to discuss any possible problems with assay interference.

The 50 tested hits were screened with a Pan assay interference compounds (PAINS) remover tool (PAINS 1, 2 and 3 filter) implemented in Canvas [16,17]. The remover tool hit on compounds 4, 26 and 27 (Figure S13), which were all tested as inactive in our experimental setup. The only active compound found by the filtering tool was compound 17 (Figure SI13), which was highlighted as a PAINS compound because it contains a quinone structure. Quinones are known for exerting unspecific redox activity that often leads them to appear as active in assays but renders their effects useless in a cellular context. To test for unspecific effects the compounds were also tested on HDAC10 and compound 17 did indeed also exert an effect. We therefore believe the effect on GCPII is unspecific and should not be further pursued.

Compound 22, a weakly active compound that represents a new symmetric scaffold previously unreported for GCPII inhibition was also highlighted as a PAINS compound because of its two 2-amino-3-carbonylthiophene groups that might cause protein thiol oxidation [18]. However, GCPII does not contain any thiol group in the proximity of the catalytic site. Moreover, this group only rarely triggers non-specific false positive readout in activity assays [16], compound 22 bears an additional aromatic substitution at position 4 of the thiophene ring, and specific inhibitors of other proteins bearing this functional group exist, such as inhibitors of tubulin assembly [19], inhibitors of tyrosin kinase FLT3 [20] or Hepatitis C virus inhibitors [21].

6. Docking Parameters for compound 46

The GCPII structure 6HKZ [22] was downloaded from PDB [2] and prepared for docking using the protein preparation wizard implemented in the Schroedinger 2020-3 small molecule drug discovery suite (SMDD) [23] using standard settings. All water molecules were deleted from the structure. The protonation states of the amino acids were calculated using Epik at pH 7 with standard settings. The protein-ligand complex was minimized with an OPLS3 force field in three steps. Initially, only hydrogens were allowed to move. In a second minimization step both hydrogens and heavy atoms were allowed to change position up to a maximum of 0.3 Å. Finally, a fully flexible minimization of the complex within the protein preparation wizard was performed.

The docking simulation was conducted using the Glide module of Schrodinger SMDD suite [24] in XP (extra precision) mode under default settings (flexible ligand sampling, OPLS3e force field, no constraints). Ten poses were calculated per ligand.

To validate the docking workflow a redocking of the co-crystallized ligand of 6HKZ was performed under these settings, resulting in an RMSD of 2.57 Å.

Glide XP [25] was employed as a scoring function, resulting in a score of -7.465 kcal/mol for compound 46 in the pose shown in Fig. 11.

7. GCPII inhibitors with subnanomolar activities

All known highly potent GCPII inhibitors share common structural motifs: A glutamate moiety binding to the S1’ pocket and either a urea or phosphonate groups that act as zinc binding locations.

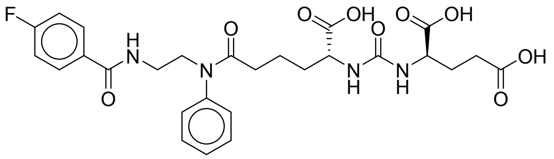

CHEMBL4286872   IC50=0.15 nM

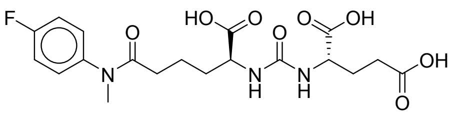

CHEMBL4288368   IC50=0.20 nM

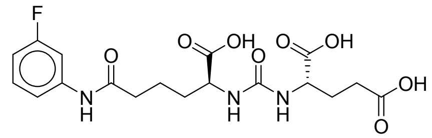

CHEMBL4277729   IC50=0.28 nM

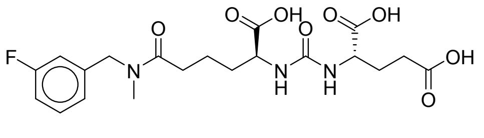

CHEMBL4291245   IC50=0.46 nM

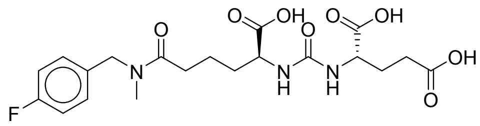

CHEMBL4283406   IC50=0.64 nM

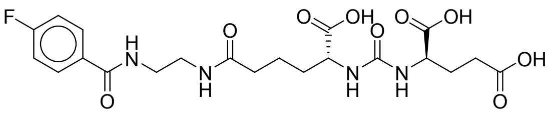

CHEMBL4294773   IC50=0.94 nM

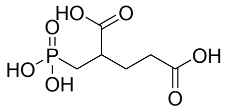

CHEMBL47009   IC50=0.30 nM

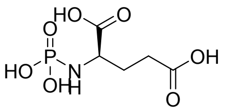

CHEMBL241779   IC50=0.86 nM

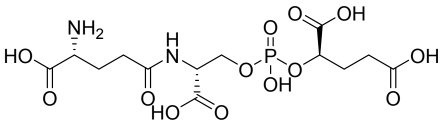

CHEMBL1289263   IC50=0.55 nM

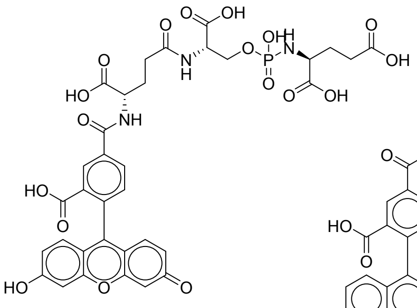

CHEMBL1921898   IC50=0.41 nM

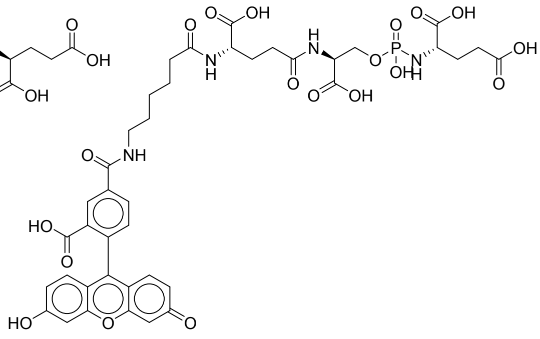

CHEMBL1921899   IC50=0.41 nM

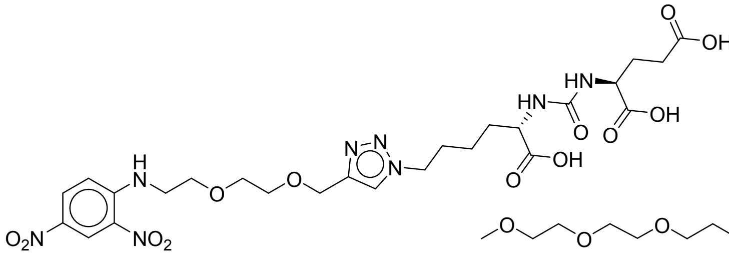

2XEI IC_{50}=0.54 nM

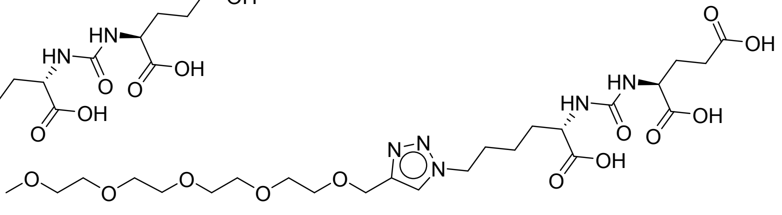

2XEJ IC_{50}=0.46 nM

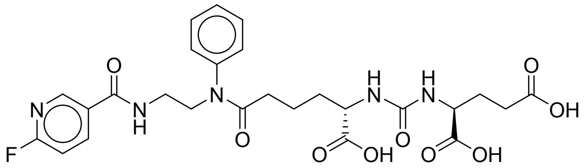

6HKZ - CHEMBL4280968 IC_{50}=0.08 nM

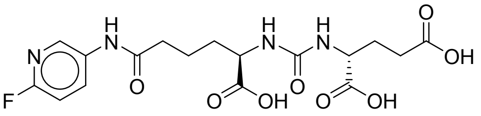

6HKJ - CHEMBL4292853 IC_{50}=0.99 nM

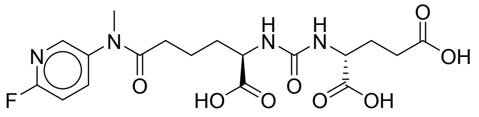

6H7Z - CHEMBL4284995 IC_{50}=0.13 nM

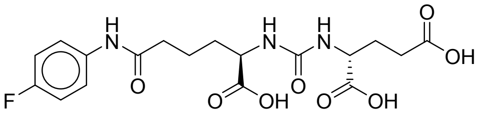

6H7Y - CHEMBL4291782 IC_{50}=0.35 nM

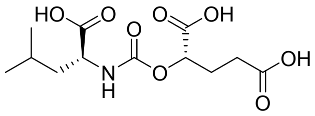

6F5L - CHEMBL4442450 IC_{50}=0.65 nM

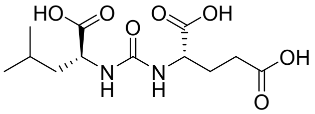

6FE5 - CHEMBL596351 IC_{50}=0.50 nM

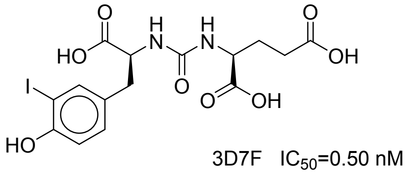

3D7F IC_{50}=0.50 nM

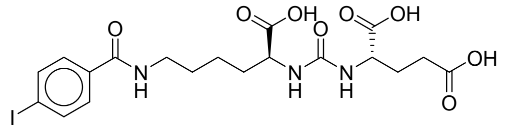

3D7H - CHEMBL510924 IC_{50}=0.06 nM

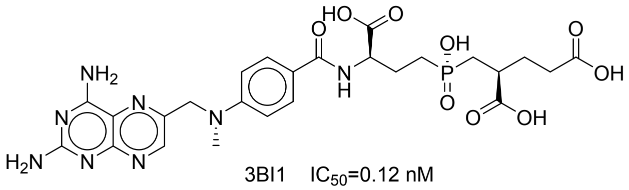

3BI1 IC_{50}=0.12 nM

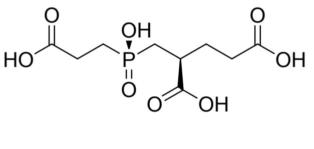

3BHX IC_{50}=0.70 nM

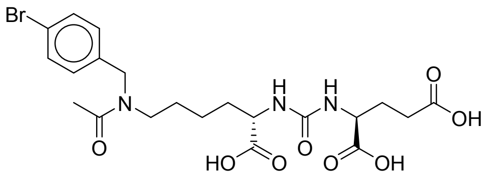

4NGM IC_{50}=0.05 nM

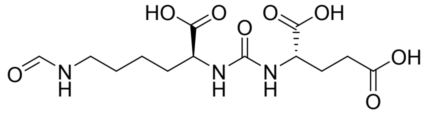

4NGN IC_{50}=0.27 nM

Fig. S20. GPCII inhibitors with subnanomolar inhibitory activities listed with either their ChEMBL ID or PDB ID where they were co-crystallized with GCPII, or both (when available). CHEMBL4286872, CHEMBL4288368, CHEMBL4277729, CHEMBL4291245, CHEMBL4283406, CHEMBL4294773 [22],

CHEMBL47009 [26], CHEMBL1289263 [27], CHEMBL241779 [28], CHEMBL1921898, CHEMBL1921899 [29], 2XEI, 2XEJ [8], 6HKZ, 6HKJ, 6H7Z, 6H7Y [22], 6F5L, 6FE5 [30], 3D7F, 3D7H [31], 3BI1, 3BHX [32], 4NGM, 4NGN [33].

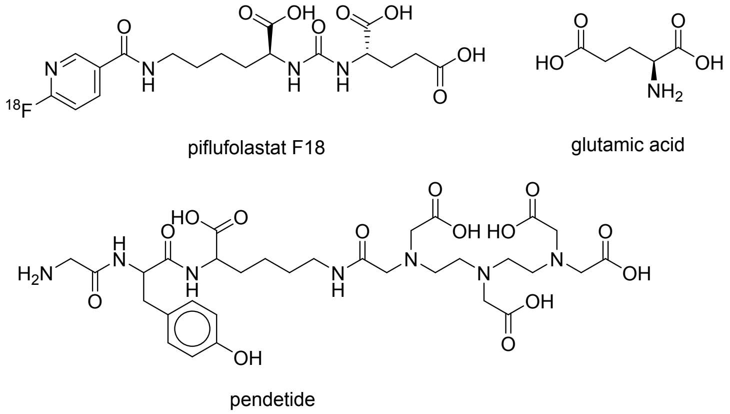

Fig. S21. FDA approved GCPII inhibitors.

similarity	cmpnd_46	cmpnd_17	cmpnd_18	cmpnd_22	cmpnd_24	cmpnd_28
cmpnd_46	1,000	0,042	0,042	0,082	0,093	0,088
cmpnd_17	0,042	1,000	0,043	0,079	0,139	0,122
cmpnd_18	0,042	0,043	1,000	0,070	0,065	0,060
cmpnd_22	0,082	0,079	0,070	1,000	0,119	0,111
cmpnd_24	0,093	0,139	0,065	0,119	1,000	0,444
cmpnd_28	0,088	0,122	0,060	0,111	0,444	1,000
4NGM	0,089	0,070	0,048	0,085	0,075	0,071
4NGN	0,039	0,091	0,028	0,063	0,069	0,065
2XEI	0,050	0,048	0,091	0,056	0,058	0,056
2XEJ	0,046	0,058	0,048	0,066	0,061	0,059
3BHX	0,037	0,125	0,035	0,066	0,098	0,089
3BI1	0,057	0,051	0,043	0,066	0,054	0,060
3D7F	0,071	0,084	0,055	0,088	0,107	0,128
3D7H	0,091	0,074	0,050	0,079	0,080	0,075
6H7Y	0,054	0,077	0,051	0,100	0,083	0,079
6H7Z	0,058	0,071	0,056	0,085	0,076	0,072
6HKJ	0,059	0,073	0,058	0,096	0,079	0,074
6HKZ	0,061	0,056	0,047	0,071	0,060	0,057
6F5L	0,043	0,111	0,033	0,083	0,086	0,079
6FE5	0,043	0,111	0,033	0,083	0,086	0,079
ChEMBL4286872	0,057	0,059	0,041	0,074	0,063	0,060
ChEMBL4288368	0,053	0,074	0,050	0,089	0,080	0,076
ChEMBL4277729	0,059	0,074	0,050	0,106	0,092	0,087
ChEMBL4291245	0,057	0,069	0,047	0,092	0,085	0,081
ChEMBL1289263	0,038	0,086	0,027	0,070	0,066	0,062
ChEMBL4283406	0,052	0,071	0,048	0,086	0,077	0,073
ChEMBL241779	0,048	0,149	0,039	0,086	0,119	0,106
ChEMBL4294773	0,050	0,067	0,046	0,082	0,072	0,069
ChEMBL47009	0,040	0,152	0,040	0,074	0,122	0,109
ChEMBL1921898	0,110	0,044	0,038	0,070	0,074	0,079
ChEMBL1921899	0,100	0,039	0,034	0,062	0,065	0,069
piflufolastat	0,057	0,070	0,056	0,076	0,075	0,071
glutamic acid	0,043	0,184	0,045	0,082	0,152	0,132
pendetide	0,046	0,059	0,041	0,074	0,063	0,069

Table S5. ECFP4 fingerprint based Tanimoto similarity between our six GCPII active compounds (blue background) and known GCPII inhibitors with subnanomolar activities from either ChEMBL database or PDB database (green background) and the three FDA approved GCPII inhibitors (orange background).

References:

(1) Wolber, G.; Langer, T. LigandScout: 3-D Pharmacophores Derived from Protein-Bound Ligands and Their Use as Virtual Screening Filters. *J. Chem. Inf. Model.* **2005**, *45* (1), 160–169. https://doi.org/10.1021/ci049885e.

(2) Berman, H. M.; Westbrook, J.; Feng, Z.; Gilliland, G.; Bhat, T. N.; Weissig, H.; Shindyalov, I. N.; Bourne, P. E. The Protein Data Bank. *Nucleic Acids Res.* **2000**, *28* (1), 235–242.

(3) Vuorinen, A.; Nashev, L. G.; Odermatt, A.; Rollinger, J. M.; Schuster, D. Pharmacophore Model Refinement for 11β-Hydroxysteroid Dehydrogenase Inhibitors: Search for Modulators of Intracellular Glucocorticoid Concentrations. *Mol. Inform.* **2014**, *33* (1), 15–25. https://doi.org/10.1002/minf.201300063.

(4) Novakova, Z.; Wozniak, K.; Jancarik, A.; Rais, R.; Wu, Y.; Pavlicek, J.; Ferraris, D.; Havlinova, B.; Ptacek, J.; Vavra, J.; Hin, N.; Rojas, C.; Majer, P.; Slusher, B. S.; Tsukamoto, T.; Barinka, C. Unprecedented Binding Mode of Hydroxamate-Based Inhibitors of Glutamate Carboxypeptidase II: Structural Characterization and Biological Activity. *J. Med. Chem.* **2016**, *59* (10), 4539–4550. https://doi.org/10.1021/acs.jmedchem.5b01806.

(5) Plechanovová, A.; Byun, Y.; Alquicer, G.; Škultétyová, L.; Mlčochová, P.; Němcová, A.; Kim, H.-J.; Navrátil, M.; Mease, R.; Lubkowski, J.; Pomper, M.; Konvalinka, J.; Rulíšek, L.; Bařinka, C. Novel Substrate-Based Inhibitors of Human Glutamate Carboxypeptidase II with Enhanced Lipophilicity. *J. Med. Chem.* **2011**, *54* (21), 7535–7546. https://doi.org/10.1021/jm200807m.

(6) Bařinka, C.; Rovenská, M.; Mlčochová, P.; Hlouchová, K.; Plechanovová, A.; Majer, P.; Tsukamoto, T.; Slusher, B. S.; Konvalinka, J.; Lubkowski, J. Structural Insight into the Pharmacophore Pocket of Human Glutamate Carboxypeptidase II. *J. Med. Chem.* **2007**, *50* (14), 3267–3273. https://doi.org/10.1021/jm070133w.

(7) Mesters, J. R.; Henning, K.; Hilgenfeld, R. Human Glutamate Carboxypeptidase II Inhibition: Structures of GCPII in Complex with Two Potent Inhibitors, Quisqualate and 2-PMPA. *Acta Crystallogr. Sect. D Biol. Crystallogr.* **2007**, *63* (4), 508–513. https://doi.org/10.1107/S090744490700902X.

(8) Zhang, A. X.; Murelli, R. P.; Barinka, C.; Michel, J.; Cocleaza, A.; Jorgensen, W. L.; Lubkowski, J.; Spiegel, D. A. A Remote Arene-Binding Site on Prostate Specific Membrane Antigen Revealed by Antibody-Recruiting Small Molecules. *J. Am. Chem. Soc.* **2010**, *132* (36), 12711–12716. https://doi.org/10.1021/ja104591m.

(9) BIOVIA, Dassault Systèmes, Discovery Studio, V18.1.100.18065, San Diego: Dassault Systèmes, 2018. 2018.

(10) Gaulton, A.; Hersey, A.; Nowotka, M. L.; Patricia Bento, A.; Chambers, J.; Mendez, D.; Mutowo, P.; Atkinson, F.; Bellis, L. J.; Cibrian-Uhalte, E.; Davies, M.; Dedman, N.; Karlsson, A.; Magarinos, M. P.; Overington, J. P.; Papadatos, G.; Smit, I.; Leach, A. R. The ChEMBL Database in 2017. *Nucleic Acids Res.* **2017**, *45* (D1), D945–D954. https://doi.org/10.1093/NAR/GKW1074.

(11) Ghose, A. K.; Crippen, G. M. Atomic Physicochemical Parameters for Three-Dimensional Structure-Directed Quantitative Structure-Activity Relationships I. Partition Coefficients as a Measure of Hydrophobicity. *J. Comput. Chem.* **1986**, *7* (4), 565–577. https://doi.org/10.1002/jcc.540070419.

(12) BIOVIA, Dassault Systèmes, BIOVIA Workbook, Release 2019; BIOVIA Pipeline Pilot, Release 2019,

San Diego: Dassault Systèmes, 2019. 2019.

(13) Tanimoto, T. T. An Elementary Mathematical Theory of Classification and Prediction | National Library of Australia. *Int. Bus. Mach. Corp.* **1958**, 1–10.

(14) Rogers, D.; Hahn, M. Extended-Connectivity Fingerprints. *J. Chem. Inf. Model.* **2010**, *50* (5), 742–754. https://doi.org/10.1021/ci100050t.

(15) Rogers, D.; Brown, R. D.; Hahn, M. Using Extended-Connectivity Fingerprints with Laplacian-Modified Bayesian Analysis in High-Throughput Screening Follow-Up. *J. Biomol. Screen.* **2005**, *10* (7), 682–686. https://doi.org/10.1177/1087057105281365.

(16) Baell, J. B.; Holloway, G. A. New Substructure Filters for Removal of Pan Assay Interference Compounds (PAINS) from Screening Libraries and for Their Exclusion in Bioassays. *J. Med. Chem.* **2010**, *53* (7), 2719–2740. https://doi.org/10.1021/jm901137j.

(17) Canvas, Version 4.5.139, Small-Molecule Drug Discovery Suite 2020-3, Schrödinger, LLC, New York, NY. 2020.

(18) Huth, J. R.; Mendoza, R.; Olejniczak, E. T.; Johnson, R. W.; Cothron, D. A.; Liu, Y.; Lerner, C. G.; Chen, J.; Hajduk, P. J. ALARM NMR: A Rapid and Robust Experimental Method to Detect Reactive False Positives in Biochemical Screens. *J. Am. Chem. Soc.* **2005**, *127* (1), 217–224. https://doi.org/10.1021/ja0455547.

(19) Morgan, R. E.; Ahn, S.; Nzimiro, S.; Fotie, J.; Phelps, M. A.; Cotrill, J.; Yakovich, A. J.; Sackett, D. L.; Dalton, J. T.; Werbovetz, K. A. Inhibitors of Tubulin Assembly Identified through Screening a Compound Library. *Chem. Biol. Drug Des.* **2008**, *72* (6), 513–524. https://doi.org/10.1111/j.1747-0285.2008.00729.x.

(20) Patch, R. J.; Baumann, C. A.; Liu, J.; Gibbs, A. C.; Ott, H.; Lattanze, J.; Player, M. R. Identification of 2-Acylaminothiophene-3-Carboxamides as Potent Inhibitors of FLT3. *Bioorganic Med. Chem. Lett.* **2006**, *16* (12), 3282–3286. https://doi.org/10.1016/J.BMCL.2006.03.032.

(21) Louise-May, S.; Yang, W.; Nie, X.; Liu, D.; Deshpande, M. S.; Phadke, A. S.; Huang, M.; Agarwal, A. Discovery of Novel Dialkyl Substituted Thiophene Inhibitors of HCV by in Silico Screening of the NS5B RdRp. *Bioorganic Med. Chem. Lett.* **2007**, *17* (14), 3905–3909. https://doi.org/10.1016/J.BMCL.2007.04.103.

(22) Nakajima, R.; Nováková, Z.; Tueckmantel, W.; Motlová, L.; Bařinka, C.; Kozikowski, A. P. 2-Aminoadipic Acid-C(O)-Glutamate Based Prostate-Specific Membrane Antigen Ligands for Potential Use as Theranostics. *ACS Med. Chem. Lett.* **2018**, *9* (11), 1099–1104. https://doi.org/10.1021/acsmedchemlett.8b00318.

(23) Small-Molecule Drug Discovery Suite 2020-3, Schrödinger, LLC, New York, NY,. 2020.

(24) Friesner, R. A.; Banks, J. L.; Murphy, R. B.; Halgren, T. A.; Klicic, J. J.; Mainz, D. T.; Repasky, M. P.; Knoll, E. H.; Shelley, M.; Perry, J. K.; Shaw, D. E.; Francis, P.; Shenkin, P. S. Glide: A New Approach for Rapid, Accurate Docking and Scoring. 1. Method and Assessment of Docking Accuracy. *J. Med. Chem.* **2004**, *47* (7), 1739–1749. https://doi.org/10.1021/jm0306430.

(25) Friesner, R. A.; Murphy, R. B.; Repasky, M. P.; Frye, L. L.; Greenwood, J. R.; Halgren, T. A.; Sanschagrin, P. C.; Mainz, D. T. Extra Precision Glide: Docking and Scoring Incorporating a Model of Hydrophobic Enclosure for Protein-Ligand Complexes. *J. Med. Chem.* **2006**, *49* (21), 6177–6196. https://doi.org/10.1021/JM051256O/SUPPL_FILE/JM051256OSI20060602_023733.PDF.

(26) Majer, P.; Jackson, P. F.; Delahanty, G.; Grella, B. S.; Ko, Y. Sen; Li, W.; Liu, Q.; Maclin, K. M.; Poláková, J.; Shaffer, K. A.; Stoermer, D.; Vitharana, D.; Yanjun Wang, E.; Zakrzewski, A.; Rojas, C.; Slusher, B. S.; Wozniak, K. M.; Burak, E.; Limsakun, T.; Tsukamoto, T. Synthesis and Biological Evaluation of Thiol-Based Inhibitors of Glutamate Carboxypeptidase II: Discovery of an Orally Active GCP II Inhibitor. *J. Med. Chem.* **2003**, *46* (10), 1989–1996. https://doi.org/10.1021/JM020515W/ASSET/IMAGES/MEDIUM/JM020515WN00001.GIF.

(27) Liu, T.; Wu, L. Y.; Hopkins, M. R.; Choi, J. K.; Berkman, C. E. A Targeted Low Molecular Weight Near-Infrared Fluorescent Probe for Prostate Cancer. *Bioorg. Med. Chem. Lett.* **2010**, *20* (23), 7124–7126. https://doi.org/10.1016/J.BMCL.2010.09.057.

(28) Wu, L. Y.; Anderson, M. O.; Toriyabe, Y.; Maung, J.; Campbell, T. Y.; Tajon, C.; Kazak, M.; Moser, J.; Berkman, C. E. The Molecular Pruning of a Phosphoramidate Peptidomimetic Inhibitor of Prostate-Specific Membrane Antigen. *Bioorg. Med. Chem.* **2007**, *15* (23), 7434–7443. https://doi.org/10.1016/J.BMC.2007.07.028.

(29) Liu, T.; Nedrow-Byers, J. R.; Hopkins, M. R.; Berkman, C. E. Spacer Length Effects on in Vitro Imaging and Surface Accessibility of Fluorescent Inhibitors of Prostate Specific Membrane Antigen. *Bioorg. Med. Chem. Lett.* **2011**, *21* (23), 7013–7016. https://doi.org/10.1016/J.BMCL.2011.09.115.

(30) Barinka, C.; Novakova, Z.; Hin, N.; Bím, D.; Ferraris, D. V.; Duvall, B.; Kabarriti, G.; Tsukamoto, R.; Budesinsky, M.; Motlova, L.; Rojas, C.; Slusher, B. S.; Rokob, T. A.; Rulíšek, L.; Tsukamoto, T. Structural and Computational Basis for Potent Inhibition of Glutamate Carboxypeptidase II by Carbamate-Based Inhibitors. *Bioorg. Med. Chem.* **2019**, *27* (2), 255–264. https://doi.org/10.1016/j.bmc.2018.11.022.

(31) Barinka, C.; Byun, Y.; Dusich, C. L.; Banerjee, S. R.; Chen, Y.; Castanares, M.; Kozikowski, A. P.; Mease, R. C.; Pomper, M. G.; Lubkowski, J. Interactions between Human Glutamate Carboxypeptidase II and Urea-Based Inhibitors: Structural Characterization. *J. Med. Chem.* **2008**, *51* (24), 7737–7743. https://doi.org/10.1021/jm800765e.

(32) Barinka, C.; Hlouchova, K.; Rovenska, M.; Majer, P.; Dauter, M.; Hin, N.; Ko, Y.-S.; Tsukamoto, T.; Slusher, B. S.; Konvalinka, J.; Lubkowski, J. Structural Basis of Interactions between Human Glutamate Carboxypeptidase II and Its Substrate Analogs. *J. Mol. Biol.* **2008**, *376* (5), 1438–1450. https://doi.org/10.1016/j.jmb.2007.12.066.

(33) Tykvart, J.; Schimer, J.; Bařinková, J.; Pachl, P.; Poštová-Slavětínská, L.; Majer, P.; Konvalinka, J.; Šácha, P. Rational Design of Urea-Based Glutamate Carboxypeptidase II (GCPII) Inhibitors as Versatile Tools for Specific Drug Targeting and Delivery. *Bioorg. Med. Chem.* **2014**, *22* (15), 4099–4108. https://doi.org/10.1016/J.BMC.2014.05.061.
